# Nanoparticle-based drug delivery systems in urologic oncology: From targeted therapy to precision theranostics

**DOI:** 10.1016/j.mtbio.2025.102585

**Published:** 2025-11-26

**Authors:** Jiayi Ma, Youlong Hai, Kun Zheng, Xiaoyong Hu, Kai Ni

**Affiliations:** a Department of Urology, Shanghai Sixth People's Hospital Affiliated to Shanghai Jiao Tong University School of Medicine, 200233, Shanghai, China; b Department of Urology, Sun Yat-sen Memorial Hospital, Sun Yat-Sen University, Guangzhou, 510289, China

**Keywords:** Urologic cancer, Nanoparticle-based drug delivery system, Targeted therapy, Theranostic strategy, Precision medicine

## Abstract

In recent years, nanoparticle-based drug delivery systems have demonstrated significant potential in the diagnosis and treatment of urologic malignancies. Common urinary system tumors, including prostate cancer, bladder cancer, and renal cell carcinoma, are characterized by high recurrence rates, strong drug resistance, and limited diagnostic sensitivity, posing substantial challenges to effective clinical management. Compared with conventional therapeutic approaches, nanoparticles—through enhanced permeability and retention (EPR) effects, surface ligand modifications, and controlled release capabilities—enable precise drug accumulation at tumor sites, thereby enhancing efficacy while minimizing systemic toxicity. This review provides a comprehensive summary of recent advances in inorganic (e.g., gold, silver, magnetic, mesoporous silica), organic (e.g., liposomes, PLGA, dendrimers), and hybrid nanoparticle platforms for urologic cancers. It highlights their multifaceted roles in targeted chemotherapy, immunotherapy, gene delivery, photothermal/photodynamic therapy, multimodal imaging, and integrated theranostic strategies. Additionally, the article critically analyzes key challenges in clinical translation, including long-term biocompatibility, immune activation, in vivo clearance, and scalable production. Finally, it discusses future directions such as stimuli-responsive nanoplatforms, personalized precision medicine, and synergistic multimodal technologies. This review aims to provide a theoretical foundation and technical reference for the clinical transformation of nanomedicine in urologic oncology.

## Graphical abstract

**Figure:**
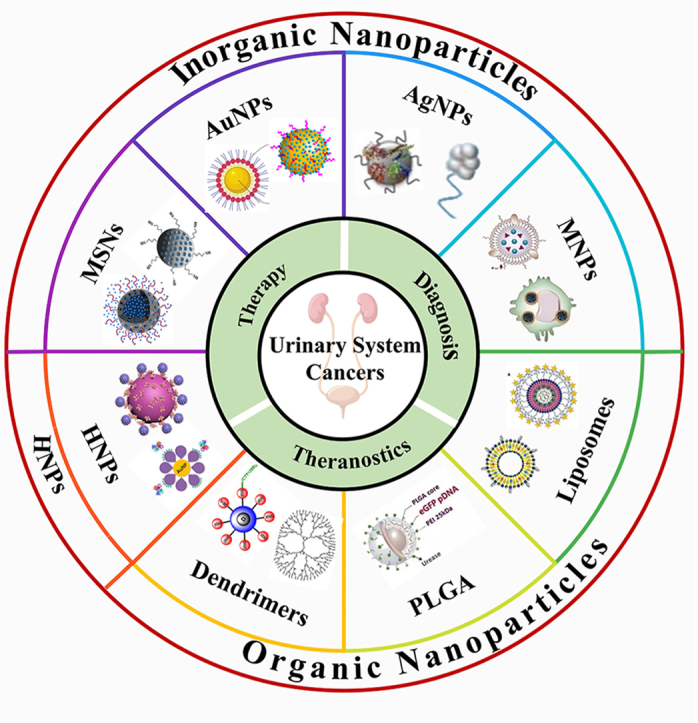

## 1 Introduction

Urinary system tumors are among the most common types of malignancies. Although advances in medical technology have continuously improved diagnostic and therapeutic approaches for these tumors, traditional treatments still face significant challenges due to their high recurrence rates and drug resistance. This is particularly evident in advanced-stage patients, whose treatment outcomes are often compromised by severe drug side effects and poor targeting, ultimately diminishing their quality of life and survival rates [1]. The use of nanoparticles as drug delivery systems can effectively overcome these limitations of conventional therapies. Nanoparticles possess unique physicochemical properties, such as small size, large surface area, and favorable biocompatibility, which enable precise targeting within the tumor microenvironment, reduce toxicity to normal tissues, and enhance drug solubility and bioavailability [2,3]. Especially in the diagnosis and treatment of urinary system tumors, nanoparticles have emerged as a critical research tool. By exploiting the enhanced permeability and retention (EPR) effect, they can accurately deliver therapeutic agents or imaging probes, thereby improving therapeutic efficacy and supporting early diagnosis [4].

**Fig. 1:**
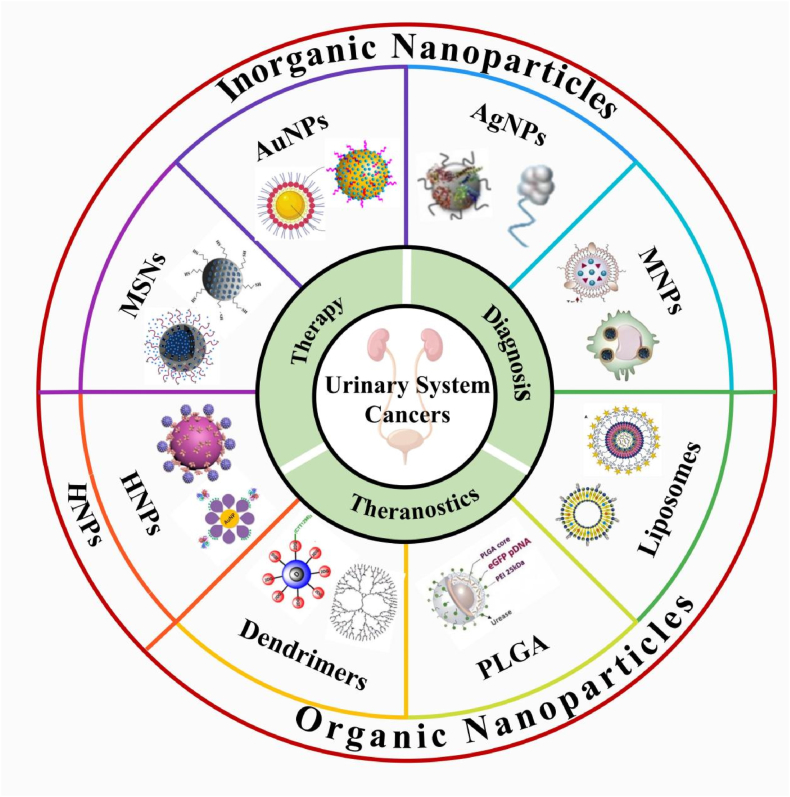
Illustration of inorganic, organic nanoparticle and HNPs-based platforms applied in the therapy, diagnosis, and theranostics of urinary system cancers.

This review provides a systematic overview of recent advances in nanoparticle-based drug delivery systems for the diagnosis and treatment of urologic cancers, with a particular focus on their applications in prostate cancer, bladder cancer, and renal cell carcinoma (Fig. 1). It highlights the advantages of inorganic, organic, and hybrid nanoparticle platforms in enhancing therapeutic specificity, overcoming drug resistance, and enabling integrated theranostic approaches such as targeted chemotherapy, gene delivery, immunotherapy, and multimodal imaging. The review further analyzes key challenges in clinical translation, including issues related to biocompatibility, long-term safety, and scalable manufacturing. Based on these insights, the article outlines future research directions, including microenvironment-responsive nanoplatforms, personalized precision medicine, and multimodal synergistic strategies, underscoring the transformative potential of nanotechnology in advancing precision oncology for urologic malignancies.

## 2 Types of urinary tumors and challenges

### 2.1 Types of urinary tumors

Cancer is one of the major social, public health, and economic challenges of the 21st century. According to the 2022 statistics from the International Agency for Research on Cancer (IARC), there were nearly 20 million new cancer cases and approximately 9.7 million cancer-related deaths worldwide [5]. Urinary system tumors mainly include prostate cancer, bladder cancer, and renal cell carcinoma (RCC), accounting for 7.3 %, 3.1 %, and 2.2 % of new cancer cases globally, and 4.1 %, 2.3 %, and 1.6 % of cancer-related deaths, respectively. Other tumors such as adrenal cancer, penile cancer, and testicular cancer are also included. The high incidence and mortality rates of these cancers present a significant burden to global healthcare systems [5,6].

(1) Prostate cancer is the second most commonly diagnosed cancer worldwide and the fifth leading cause of cancer-related death in men, with approximately 1.5 million new cases and 397,000 deaths reported globally [5]. In recent years, the incidence of prostate cancer has risen rapidly due to aging populations and dietary changes, severely impacting the quality of life of middle-aged and elderly men [7,8]. The development of prostate cancer is closely associated with genetic factors (such as BRCA1/2 mutations) and hormonal influences (notably androgen activity). Its progression involves multiple biological processes, including epithelial-mesenchymal transition (EMT), angiogenesis, invasion, and metastasis [9].
(2) Bladder cancer is the seventh most common cancer in men and ranks seventeenth among all cancers worldwide [10]. Its incidence varies across regions, likely due to differing levels of exposure to environmental carcinogens. Established risk factors include smoking and occupational exposures, while other factors such as diet, gene-environment interactions, and pelvic radiation therapy also contribute to disease development [11]. Globally, the incidence and mortality of bladder cancer are on the rise, with particularly noticeable increases in developed countries [10].
(3) Renal cell carcinoma (RCC) is a malignant tumor originating from the epithelial cells of the renal tubules and accounts for approximately 90 % of all malignant kidney tumors. It is the most common solid lesion of the kidney [12]. Among RCC subtypes, clear cell RCC (ccRCC) constitutes about 70 %, papillary RCC (pRCC) accounts for 10 %–15 %, and chromophobe RCC (chRCC) about 5 % [13,14].RCC typically has an insidious onset, is highly malignant, and progresses rapidly. Early-stage RCC often presents no obvious symptoms, with clinical manifestations usually appearing only in advanced stages. The classical triad of flank pain, hematuria, and abdominal mass is infrequently observed [15]. RCC is often discovered incidentally during imaging for other abdominal conditions or nonspecific symptoms, and approximately 17 % of patients are diagnosed at a stage with distant metastases [16].
(4) Other types of urologic tumors include penile cancer, adrenal cancer, and testicular cancer. Penile cancer is usually squamous cell carcinoma with early lymphatic spread. Adrenal tumors such as adrenocortical carcinoma and pheochromocytoma may cause hormone-related symptoms. Testicular cancer, common in young men, typically presents as a painless mass and has high cure rates when treated early [5].

### 2.2 Challenges of conventional treatment and diagnosis methods

Chemotherapy and radiotherapy remain the primary treatments for urinary system tumors. However, beyond heterogeneity, resistance, and toxicity, the gut microbiome also modulates tumorigenesis and treatment response, further impacting efficacy and quality of life [17,18].

(1) Androgen receptor (AR) signaling is pivotal in prostate cancer progression [19]. Androgen deprivation therapy (ADT), which inhibits testosterone synthesis to block AR signaling, is the standard approach for advanced disease [8,20]. Yet, its efficacy is limited, and most patients eventually develop castration-resistant prostate cancer (CRPC), which can progress into metastatic CRPC (mCRPC) despite low androgen levels [21]. Treatments for CRPC include second-generation hormone therapies (e.g., abiraterone, enzalutamide) and chemotherapy (e.g., docetaxel, cabazitaxel). Despite recent progress, therapeutic outcomes for advanced CRPC remain suboptimal due to limited specificity and toxicity of current options [[22], [23], [24], [25]]. Hence, identifying new targets and delivery strategies is essential.
(2) Bladder cancer treatment varies with tumor stage [26]. Non-muscle-invasive bladder cancer (NMIBC) is mainly managed with transurethral resection and intravesical instillation [27,28]. Agents like mitomycin C, gemcitabine, and BCG are commonly used [[29], [30], [31]]. However, recurrence occurs in up to 40 % of NMIBC cases, with about 10 % progressing to muscle-invasive bladder cancer (MIBC) [32]. MIBC, accounting for 25–30 % of bladder cancers, has a poorer prognosis [33]. Radical cystectomy (RC) with lymph node dissection remains the standard, but RC may impair urinary, reproductive, and gastrointestinal functions [34,35]. Its applicability is further limited in elderly patients due to comorbidities [36].
(3) Renal cell carcinoma (RCC) is largely resistant to chemo- and radiotherapy, with nephrectomy as the main treatment. Nonetheless, up to 30 % of patients experience recurrence or metastasis [37]. The 5-year survival rate for advanced RCC is below 20 %, and most patients are not surgical candidates [38]. Targeted therapies combined with PD-1 inhibitors are first-line options for advanced clear cell RCC [39]. Although these treatments extend survival, their benefit varies due to RCC's pathological complexity [40]. Early detection and more effective therapies are still urgently needed.
(4) Other types of urologic tumors have varied treatments; testicular cancer has high cure rates, while penile and adrenal cancers face poor outcomes in advanced stages [41].

Nanoparticle-based drug delivery systems in nanomedicine offer targeted delivery, reduced side effects, and improved efficacy against drug-resistant tumors, making them a promising strategy in oncology.

## 3 Nanomedicine: A new era in the management of urinary system tumors

### 3.1 The potential of nanomedicine in cancer diagnosis and therapy

Nanomedicine employs engineered nanoscale materials to build diagnostic and therapeutic platforms. Compared to conventional therapies, it offers advantages in early detection, precision treatment, and safety [42]. Nanoparticles, as core carriers in nanomedicine, can be engineered to deliver drugs, photothermal agents, contrast agents, or immunomodulators. With enhanced permeability and retention (EPR) effects and surface ligand modifications, they enable targeted delivery while mimizing off-target toxicity and side effects [43,44]. The advancement of nanotechnology has introduced new opportunities for cancer diagnosis and treatment [45]. Liposomes were first introduced as anticancer drug carriers in the 1960s–70s, marking the advent of nanomedicine in oncology [46,47]. The approval of liposomal doxorubicin (Doxil) in 1995 further demonstrated its potential to enhance drug efficacy while reducing toxicity [48]. Since then, a variety of nanoparticle-based drug delivery systems have been developed, including gold nanoparticles, polymeric nanoparticles, and dendrimers, which can be tailored to meet specific tumor characteristics [49]. Moreover, the emergence of stimuli-responsive and targeted nanomedicine has opened a new era of precision oncology [50].

Beyond therapy, nanoparticles also serve as diagnostic tools. Functionalized nanoparticles carrying diagnostic agents allow for early and accurate tumor detection [51]. They can act as contrast agents to improve imaging modalities such as MRI and PET, enhancing contrast and localization accuracy [52]. Additionally, multifunctional nanoplatforms that integrate imaging agents (e.g., gold nanoparticles for CT or iron oxide for MRI) with therapeutic payloads (e.g., chemotherapeutics, siRNA) enable real-time monitoring and image-guided therapy [53].

### 3.2 Clinical translation of nanomedicine in urologic oncology

Urinary system tumors are characterized by high incidence, mortality, and recurrence rates, necessitating more precise drug delivery strategies to minimize damage to normal tissues [54]. Surface-modified nanoparticles equipped with targeting ligands can selectively bind to cancer cells, thereby reducing the off-target toxicity commonly associated with traditional chemotherapies—an especially valuable feature in urologic oncology.

One key challenge in treating these tumors lies in overcoming biological barriers. For instance, renal clearance rapidly eliminates therapeutic agents, limiting their accumulation at tumor sites. Nanoparticle encapsulation can prevent this, prolong circulation time, and enhance retention within tumor tissue [55,56]. Furthermore, the high heterogeneity of tumors such as renal cell carcinoma (RCC) can hinder uniform drug distribution. Stimuli-responsive nanocarriers can adapt to the tumor microenvironment, enabling controlled and targeted drug release. Nanomedicine also aligns well with personalized medicine, allowing treatments to be tailored to individual genetic and pathological profiles, thereby improving efficacy and minimizing adverse effects [50]. In prostate cancer, for example, nanotechnology may aid in distinguishing aggressive from indolent forms, facilitating more precise treatment decisions. Resistance to therapy—especially in RCC—remains a major issue; nanomedicine can help mitigate resistance through sustained and localized drug release [50].

From a diagnostic perspective, the nonspecific nature of early symptoms in urinary tumors highlights the need for more accurate detection. Nanoparticle-based contrast agents can enhance imaging modalities like MRI and CT, improving early tumor detection and characterization [57].

**Fig. 2:**
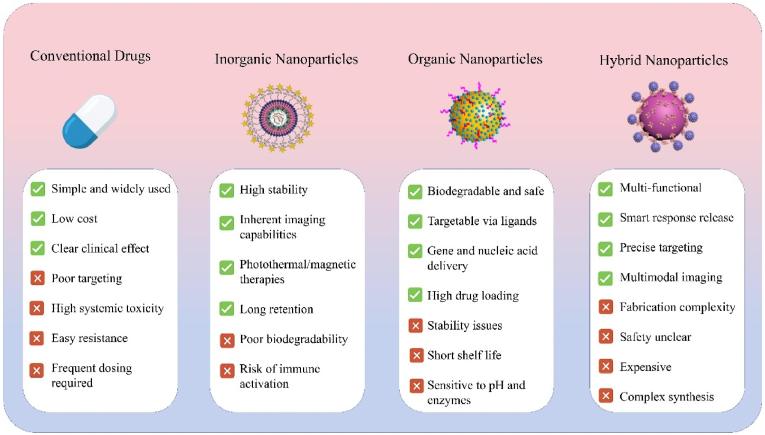
Advantages and disadvantages of conventional drugs, inorganic nanoparticles, organic nanoparticles, and hybrid nanoparticles. Reproduced with permission from Ref. [71]. [204]. [302].

In summary, nanomedicine offers significant potential in precise drug delivery, personalized therapy, diagnostic enhancement, and overcoming drug resistance in urinary system tumors. This review outlines recent advances in the application of inorganic nanoparticles (e.g., gold nanoparticles, silver nanoparticles, magnetic nanoparticles, and mesoporous silica nanoparticles), organic nanoparticles (e.g., liposomes, dendrimers, PLGA), and hybrid nanoparticles in the diagnosis and treatment of these cancers (Fig. 2).

## 4 Inorganic nanoparticles

### 4.1 Gold nanoparticles

Gold nanoparticles (AuNPs) are among the most extensively developed nanomaterials for cancer diagnosis and therapy [58]. Their unique optical properties—such as surface plasmon resonance—combined with excellent colloidal stability and versatile surface functionalization (e.g., biomolecule conjugation), make them highly suitable for early detection and targeted treatment of urinary system tumors (Table 1). Therapeutically, their high photothermal conversion efficiency enables effective tumor ablation via photothermal therapy (PTT) [59]. Through the formation of stable Au–S bonds, AuNPs can be easily functionalized with a wide range of biomolecules, including drugs, targeting ligands, and nucleic acids, allowing for high drug-loading capacity and efficient delivery [60]. Moreover, AuNPs can induce immunogenic cell death, triggering anti-tumor immune responses that suppress tumor growth and recurrence [61]. Their biocompatibility and low toxicity make them excellent candidates for combined chemo- and photothermal therapies [62].

**Table 1:** Therapeutic applications of gold nanoparticles in urologic tumors.

	Nanoparticle	Nanoparticle Size(nm)	Outcome	Cell Lines	Stage	Ref.
PC	PDA-GNRs	95	Enhanced colloidal stability and biocompatibility.	DU-145, PC3 cells	in vitro	64
	PTX-PP@Au NPs	147 ± 1.16	Synergistic PTT/PDT/CT therapy achieved under single 808 nm irradiation.	PC3 cells	in vivo in vitro	65
	AuNP-S-AB	14.5	Enhanced biocompatibility of AuNP-S-AB in cancer cells.	HepG2,PNT-2 cells	in vitro	66
	YF-AuNPs	14 ± 5	Modulate the tumor immune microenvironment; Pro-tumor macrophage targeting.	MDAMB-231, PC-3 cells	in vitro	67
	PS-AuNP	12	Utilizing PS-AuNPs to disrupt tumor PS-mediated immune evasion and enhance apoptotic signaling pathways.	LNCaP,PC3,DU-145,C4-2b cells	in vitro	68
	RALA-AuNP	110	RALA functionalization enhanced AuNP uptake by more than threefold; Significant radiosensitization, with a DEF of 1.54	PC-3,DU145 cells	in vitro	69
	Au–Gd(III)-PSMA NPs	7.8	Synergistic effect of Au and Gd(III) improved tumor suppression	PC3pip,PC3flu, LNcap cells	in vivo in vitro	71
BC	Zr(Cu)-MOF@Au@DHA	212.5	Integrating photodynamic and photothermal therapies; Synergistic photodynamic and ferroptosis mechanisms	T24 cells	in vivo in vitro	79
	PAA4 and PAA5	–	GSH-triggered Au(I) ion release	HUVEC, EJ cells	in vitro	80
	GNP-LLO91–99	1.5 ± 0.5	GNP-LLO91–99 nanovaccines significantly activate immune responses and reduce bladder tumor burden.	T-24 cells	in vivo in vitro	81
RCC	CWAuNPs	286.5	The green-synthesized gold nanoparticles exhibit low toxicity and excellent anticancer effects	A498,Sw-156 cells	in vitro	87
	GNR	44∗11	TPL microscopy provides sub-micron resolution to accurately quantify the accumulation of AuNPs	ATCC CRL-1932 cells	in vivo in vitro	88
	AuNRs-PEG	44∗11	The first simultaneous, non-invasive real-time monitoring of nanoparticle concentration and tissue hemodynamics.	–	in vivo	89

In diagnostics, AuNPs can be functionalized with targeting ligands for selective recognition of tumor markers and serve as enhanced contrast agents in multimodal imaging techniques such as photoacoustic imaging and Raman spectroscopy (Table 2). Novel AuNP-based composites—such as nanoshells and nanostars—engineered through plasmonic and computational design strategies, have demonstrated clinical potential in molecular imaging, real-time intraoperative guidance, and the broader shift of nanomedicine toward precision, personalization, and minimally invasive interventions [63].

**Table 2:** Diagnostic and theranostic applications of gold nanoparticles in urologic tumors.

	Nanoparticle	Nanoparticle Size(nm)	Outcome	Cell Lines	Stage	Ref.
PC	AG-Gd@PSMA1 NPs	3.2	AGGP enabled high-specificity MRI/CT/NIRF imaging; Achieved MPS evasion and renal clearance	PC3pip,PC3flu cells	in vivo in vitro	72
	SERS nanotags	60–70	SERS-based microfluidic sensor using a nanocone array; Enhanced SERS signal via a gold-coated nanocone array.	–	in vitro	73
	SERS nanotags	65	Enhanced detection sensitivity and multiplex capability using core-shell SERS nanotags	–	in vitro	74
	rGO-MWCNT/AuNPs	65.2	Achieved an ultra-low PSA detection limit of 1.0 pg/mL	–	in vitro	75
	H-Au	100	A label-free, real-time SPRi biosensor; Demonstrated correlation between SPRi signal and clinical t-PSA values	LNcap cells	in vitro	76
	Bt-AuNP-G	19	Demonstrated robust 5hmC quantification across various biological samples	C4-2B, RWPE-1 cells	in vivo in vitro	77
BC	GNRs@PEG-Iso4	42 × 13	Achieved stable, non-aggregating, and α5β1-targeted PEGylated GNRs	MB49 cells	in vivo in vitro	82
	AuHNR@MnO2@CS	–	Enhanced TME-triggered PTCE for selective and efficient NIR-II PTT;	MB49 cells	in vivo in vitro	83
	GNRs@Chit-Iso4	–	Simultaneous high-resolution imaging and tumor-selective photothermal ablation.	MB49 cells	in vivo in vitro	84
	GNRs@Chit-Apt-Itg	88.2 ± 6.4	Developed a urine-stable aptamer-conjugated GNRs nanoplatform for submillimeter bladder cancer detection.	T24 、 RT112 、 MB49-Luc cells	in vitro	85
	GNRs@Chit-*Iso4*	90.2 ± 7.2	Ultrasound-assisted shaking enhances imaging efficiency and stability.	RT4,5637,HT-1376,RT112 cells	in vivo in vitro	86
RCC	AuNPs	–	The PS-LAMP method achieves highly sensitive and quantitative ctDNA methylation detection	–	in vitro	90
	AuNPET-LDI MS	–	Apply AuNPET-SALDI MS technology for non-invasive metabolomic profiling of different RCC types and stages,	–	in vitro	91
Others	AuNPs-ARG	5.5 ± 1.1	Label-free AuNPs-ARG immunosensor efficiently detects DHEAS biomarker for early diagnosis.	–	in vitro	92
	GNP	30	Dual-ligand AuNP probe enables visual NMN detection in urine with 0.5 μM sensitivity.	–	in vitro	93

#### 4.1.1 Targeted therapeutics and photothermal synergy in prostate cancer

**Fig. 3:**
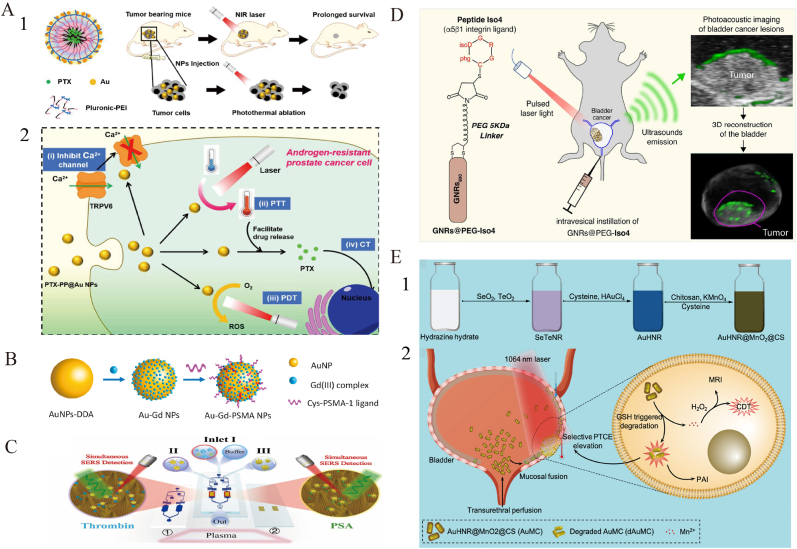
**Multimodal diagnostic and therapeutic strategies of AuNPs in urinary system tumors. A:** Schematic of gold-caged copolymer nanoparticles for combined chemo-photo therapy in androgen-resistant prostate cancer (ARPC). (1) Nanoparticles accumulate in tumors via EPR and induce therapeutic effects upon NIR irradiation. (2) Mechanism includes TRPV6 inhibition, photothermal therapy (PTT), paclitaxel (PTX) release for chemotherapy (CT), and reactive oxygen species (ROS) generation for photodynamic therapy (PDT). Reproduced with permission from Ref. [65].**B:** Au–Gd(III)-PSMA nanoparticles for MR-guided radiotherapy. Design of AuNP core stabilized by Gd(III) complexes and targeted by Cys-PSMA-1 ligand. [71].**C:** (1) Schematic illustration of the SERS-based microfluidic aptamer sensor for simultaneous immunoassay of dual prostate cancer biomarkers. [73]. **D:** (1) Synthesis Route of AuHNR@MnO2@CS. 2) TME GSH Responsive Elevation of Photothermal Conversion Efficiency (PTCE) for Precise Therapy of Orthotopic Bladder Cancer. Reproduced with permission from Ref. [83]. **E:**GNRs@PEG-Iso4 represents a simple and robust contrast agent for photoacoustic imaging and diagnosis of small bladder cancer lesions. Reproduced with permission from Ref. [84]^.^ (For interpretation of the references to color in this figure legend, the reader is referred to the Web version of this article.)

Gold nanoparticles (AuNPs) are particularly well suited for prostate cancer (PCa) diagnosis and therapy for their tunable photonic behavior, high-Z radiosensitization, and ligand-friendly surfaces align well with PCa-specific targets and clinical treatment pathways [63]. Mahmoud et al. [64] developed polydopamine-coated gold nanorods (PDA-GNRs), which enhanced colloidal stability and significantly inhibited migration and adhesion of DU-145 PCa cells, indicating potential for metastasis suppression. Polymer–AuNP hybrid particles (PTX-PP@Au NPs) demonstrated a synergistic approach combining photothermal therapy (PTT), photodynamic therapy (PDT), and chemotherapy (CT), while suppressing TRPV6 channels to enhance tumor targeting and reduce systemic toxicity (Fig. 3A) [65]. Stolarczyk et al. [66] synthesized abiraterone-modified AuNPs (AuNP-S-AB) via ligand exchange to improve stability and biocompatibility in prostate epithelial cells (PNT-2), showing promising therapeutic potential.

Green-synthesized AuNPs using Yucca filamentosa extract (YF-AuNPs) leveraged EPR effects and receptor-mediated endocytosis to induce apoptosis and regulate cytokine expression, suggesting immunomodulatory anti-cancer mechanisms [67]. Phosphatidylserine-modified AuNPs (PS-AuNPs) induced morphological changes and DNA fragmentation in PCa cells through immunological recognition of apoptotic markers [68]. Bennie et al. [69] demonstrated that RALA-peptide-functionalized AuNPs (RALA-AuNPs) significantly increased cellular uptake and enhanced radiosensitivity in PCa cells without notable toxicity, offering a novel radiosensitization strategy. Prostate-specific membrane antigen (PSMA), a key PCa biomarker [70], was used to functionalize AuNPs with Gd(III) complexes and PSMA ligands, improving both MRI imaging contrast and radiotherapy efficacy through precise tumor targeting and dose minimization (Fig. 3B) [71].

In imaging and biomarker detection, AuNPs have demonstrated great promise. Wang et al. [72] developed a PSMA-targeted MRI/CT/NIRF trimodal imaging probe (AGGP) that enabled precise tumor localization, immune evasion, and renal clearance. Lu et al. [73] introduced a microfluidic Surface-Enhanced Raman Scattering (SERS) sensor based on Au nanocone arrays for ultrasensitive detection of PSA and thrombin (limits of detection: 0.01 ng/mL and 0.01 nM, respectively) (Fig. 3C). Chen et al. [74] further optimized vertical flow analysis (VFA) with core–shell SERS nanoprobes for multiplexed detection of PSA, CEA, and AFP with detection limits of 0.37, 0.43, and 0.26 pg/mL, respectively.

An rGO-MWCNT/AuNP composite electrochemical aptasensor achieved PSA detection down to 1.0 pg/mL with excellent repeatability [75]. For exosome analysis, H-Au supramolecular Surface Plasmon Resonance Imaging (SPRi) biosensors sensitively identified PCa-derived exosomes and showed strong correlation with clinical t-PSA results [76]. Additionally, a biotin-AuNP-based electrochemical sensor enabled label-free detection of 5-hydroxymethylcytosine (5hmC) with a detection limit of 63.2 fM, offering a powerful tool for early cancer diagnosis and prognosis [77].

#### 4.1.2 Gold nanoparticles for bladder cancer: enhancing detection and delivery

Gold nanorods (GNRs), widely applied in photothermal therapy (PTT), have shown—via simulation studies—that uniform distribution significantly enhances ablation efficiency while minimizing collateral tissue damage [78]. A GNR-modified metal–organic framework (Zr(Cu)-MOF@Au@DHA) was developed to integrate PTT, photodynamic therapy (PDT), and ferroptosis. It enhances reactive oxygen species generation, depletes glutathione, and releases dihydroartemisinin (DHA), resulting in improved therapeutic outcomes [79]. Additionally, AuNPs combined with ultra-carbon center gold clusters (PAA4 and PAA5) can release Au(I) ions in response to glutathione, inducing ferroptosis and suppressing tumor growth with low systemic toxicity [80]. A gold nanoparticle-based vaccine (GNP-LLO91–99), conjugated with a Listeria-derived peptide, activated dendritic cells and promoted immunogenic tumor cell death, showing synergy with immune checkpoint inhibitors for bladder and other cancers [81].

For theranostics, Armanetti et al. [82] developed GNR-based probes combining photoacoustic imaging (PAI) and PTT. These probes remain stable in urine, specifically target bladder cancer cells, and detect sub-millimeter lesions. Upon laser activation, GNRs induce localized thermal damage, promoting tumor necrosis and improving survival. Another GNR-based probe, AuHNR@MnO_2_, responds to low glutathione (GSH) levels by degrading its MnO_2_ coating and enhancing NIR-II photothermal effects. Simultaneously, it releases Mn^2+^ for MRI and chemodynamic therapy (CDT) (Fig. 3D) [83].

In early diagnosis, functionalized AuNPs targeting α5β1 integrins offer promising strategies for detecting early-stage bladder cancer. PEGylated GNRs (GNRs@PEG-Iso4) achieved high-resolution PAI detection of tumors <1 mm, while RNA aptamer–GNR hybrids demonstrated strong urinary stability and sensitivity for in vivo detection of occult lesions (Fig. 3E) [84,85]. For targeted imaging, GNRs combined with Iso4 peptide and chitosan (Chit), assisted by ultrasound agitation, achieved uniform intravesical distribution and effective detection of tumors <0.5 mm via photoacoustic imaging—overcoming the limitations of conventional imaging modalities [86].

#### 4.1.3 Theranostic applications of AuNPs in renal cell carcinoma

Gold nanoparticles (GNPs) have been extensively studied in the treatment of renal cell carcinoma (RCC) due to their biocompatibility and multifunctionality. Liu et al. [87] synthesized GNPs (CWAuNPs) using the traditional Chinese herb Curcuma wenyujin, which significantly induced apoptosis in RCC cells by upregulating Caspase-3, -9, Bid, and Bad, while downregulating Bcl-2 and Bcl-xl, demonstrating therapeutic potential.

In nanoparticle delivery monitoring, two-photon luminescence (TPL) microscopy was employed to quantitatively assess gold nanorod (GNR) accumulation in cells and tissues. Morales et al. [88] established a calibration protocol correlating TPL signal intensity with GNR concentration and validated it in 786-O cells and RCC mouse models. The results closely matched those obtained from inductively coupled plasma mass spectrometry (ICP-MS), confirming its precision. Additionally, near-infrared diffuse reflectance spectroscopy (DRS) and diffuse correlation spectroscopy (DCS) were applied for non-invasive, real-time tracking of PEGylated GNRs (AuNRs-PEG) in a ccRCC model. This technique accurately quantified in vivo nanoparticle levels and simultaneously monitored tissue hemodynamics, aligning well with ICP-MS results and showing strong clinical translation potential [89].

AuNPs have also proven valuable in non-invasive RCC diagnostics. A PS-LAMP (phosphorothioate primer-based loop-mediated isothermal amplification) method combined with a one-step strand displacement (OSD) probe enabled ultrasensitive detection of methylated ctDNA, while AuNP-based colorimetric analysis provided quantitative readouts from urine and plasma samples of bladder cancer patients, suggesting a promising non-invasive diagnostic tool [90]. In metabolomic applications, AuNP-enhanced target (AuNPET) surface-assisted laser desorption/ionization mass spectrometry (SALDI-MS) was used to identify serum and urine metabolic biomarkers, supporting subtype classification and progression assessment of clear cell RCC (ccRCC), and offering new strategies for early diagnosis and prognosis [91].

#### 4.1.4 Applications of AuNPs in other types of urinary system tumors

Gold nanoparticles (AuNPs) also show high sensitivity and versatility in diagnosing other urological tumors, such as adrenal cancer. Targeting the pediatric adrenocortical carcinoma (pACC) biomarker DHEAS, Lima et al. [92] developed a label-free impedance immunosensor based on arginine-functionalized AuNPs, achieving a detection limit as low as 7.4 μg/dL. The sensor demonstrated excellent stability, specificity, and strong agreement with clinical results in real plasma samples. In another study, a colorimetric sensing system utilizing a dual-ligand recognition mechanism was constructed to detect the pheochromocytoma marker NMN. By inducing AuNP aggregation for visual readout, the system achieved a detection limit of 0.5 μM and effectively distinguished structurally similar compounds, highlighting its potential for rapid and early diagnosis [93].

### 4.2 Silver Nanoparticles

Silver nanoparticles (AgNPs) have demonstrated broad potential in cancer diagnosis and therapy due to their distinctive physicochemical properties (Table 3). Studies have shown that AgNPs possess intrinsic antiproliferative effects and can be synthesized via green methods with high stability and low cost [94,95]. While elemental silver offers good biocompatibility and low toxicity, it has poor bioavailability. In contrast, AgNPs can be internalized by cells through endocytosis, enabling localized release of Ag^+^ ions at the target site to enhance anticancer efficacy [96,97].Their antitumor mechanisms are multifaceted, involving disruption of cancer cell ultrastructure, induction of reactive oxygen species (ROS), DNA damage, and subsequent apoptosis or necrosis [98,99]. Additionally, AgNPs can modulate key genes and signaling pathways, induce cell cycle arrest, and inhibit tumor cell migration and angiogenesis, thereby reducing the risk of metastasis [100]. AgNPs have also shown potential in overcoming multidrug resistance (MDR) in tumor cells [101].

**Table 3:** Therapeutic applications of sliver nanoparticles in urologic tumors.

	Nanoparticle	Nanoparticle Size(nm)	Outcome	Cell Lines	Stage	Ref.
PC	AgNPs-PLE	15 ± 5	AgNPs-PLE induced apoptosis and cell cycle arrest in DU145 cells with low toxicity to normal cells.	DU145 cells	in vitro	102
	G-AgNP	64 ± 11	G-AgNPs induce ROS production and mitochondrial dysfunction, leading to S-phase arrest and apoptosis.	PC-3,LNCaP cell	in vitro	103
	AgNPs	100	PVP/PH-stabilized AgNPs show ∼50 times higher anticancer activity than reported formulations.	SKOV-3,PC-3,SH-SY5Y cells	in vitro	104
	AgNPs	14	PVP coating enhances nanoparticle stability and nanoparticles disrupt cancer cell migration.	PC-3 cells	in vitro	105
	AgNPs	3	Green-synthesized, starch-capped AgNPs show potent cytotoxicity against hormone-sensitive cancer cells.	LNCaP,PC-3 cells	in vitro	106
	AgNPs	21.31 ± 0.43	Silver nanoparticles combined with polyphenols from Cynara scolymus L. flower exhibit strong cytotoxicity	PC-3,A549 cells	in vitro	107
BC	AgNP	–	Biogenic silver nanoparticles demonstrated potent anti-bladder cancer activity.	5637 cells	in vivo in vitro	113
	PVP-AgNPs	30–50	AgNPs induce apoptosis by generating ROS, up-regulating Bax, down-regulating Bcl-2, and activating caspases 3 and 7.	5637 cells	in vitro	114

In diagnostics, AgNPs’ strong optical absorption and scattering properties enable their application in photoacoustic imaging. When targeted to tumor sites—particularly leaky tumor vasculature—AgNPs enhance imaging contrast, allowing effective differentiation between malignant and normal tissues [100] (Table 4).

**Table 4:** Diagnostic and theranostic applications of sliver nanoparticles in urologic tumors.

	Nanoparticle	Nanoparticle Size(nm)	Outcome	Cell Lines	Stage	Ref.
PC	Peptide-templated AgNPs	5	CCY-LWYIKC enables antibody-free, ultrasensitive PSMA-positive exosome detection.	LNCaP, MCF-7, SMMC-7721 cells	in vitro	108
	AgNPs-VB2-Ab2	22	An “on-off” ECL immunosensor based on ECL resonance energy transfer was developed.	–	in vitro	109
	PDMS@AgNPs@ZIF-67	300	Electromagnetic-chemical synergistic enhancement was achieved in a novel biomimetic substrate.	–	in vitro	110
	CS-AgNPs-Lu	–	Green electrodeposition strategy enhances ECL performance and dual-function AgNPs.	–	in vitro	111
	mucilage-GNPs-SNPs/GCE	116.9	Green nanocomposite enhances electron transfer and ultra-sensitive label-free immunosensing	–	in vitro	112
BC	AgNPs	9.73 ± 1.70	A nanoparticle-assisted SWATH-MS approach identified significant serum protein biomarkers for NMIBC	–	in vitro	115
RCC	AgNPs	30.059 ± 0.67	A 3D SERS substrate with porous membranes and AgNPs significantly enhanced cancer detection sensitivity	–	in vitro	116
Others	AgNPs	–	AgNPs-enhanced SERS enables noninvasive functional classification of adrenal tumors with 96.8 % accuracy.	–	in vitro	117

#### 4.2.1 Antitumor effects of AgNPs in prostate cancer: mechanisms and prospects

**Fig. 4:**
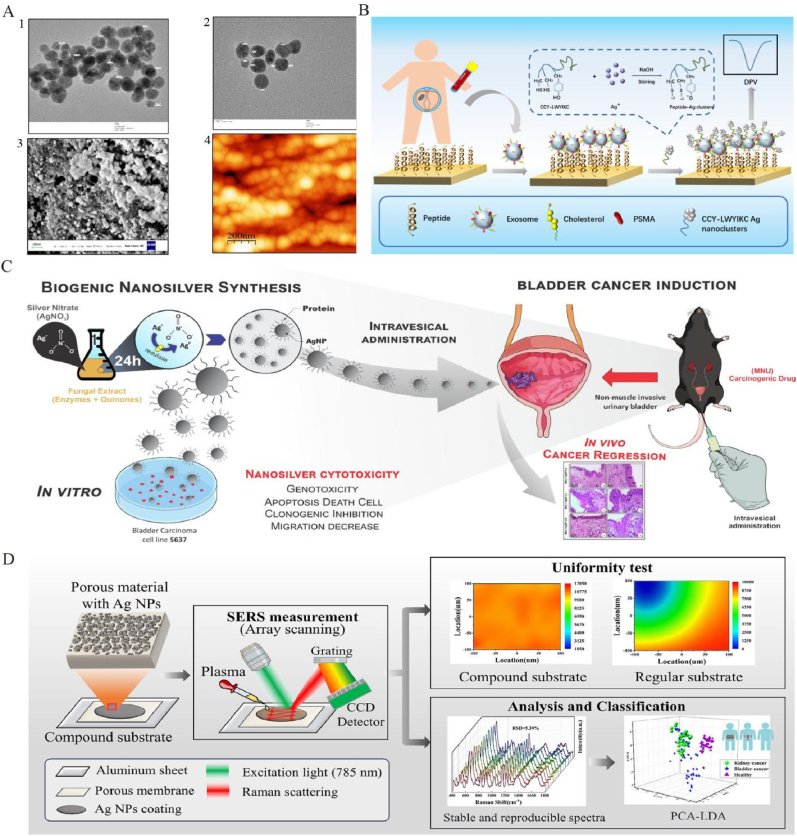
**Innovation of AgNPs in urinary system tumors. A:** Characterization of AgNPs-PLE: (1,2) TEM images showing morphology; (3) SEM micrograph indicating surface structure; (4) STM image illustrating particle topology. Reproduced with permission from Ref. [102]. **B:** Fabrication and principle of the electrochemical biosensor based on peptide-templated AgNPs nanoprobe. Reproduced with permission from Ref. [108].**C:** Schematic of biogenic nanosilver synthesis using fungal extract and intravesical application for bladder cancer therapy, highlighting both in vitro cytotoxicity mechanisms (genotoxicity, apoptosis, clonogenic inhibition, migration decrease) and in vivo cancer regression effects in NMIBC model. Reproduced with permission from Ref. [113].**D:** Schematic of a porous AgNP substrate enhancing SERS signals via nanoparticle aggregation. Uniformity tests confirmed stable signal hotspots. PCA-LDA models distinguished kidney cancer patients from healthy controls (accuracy: 98.5 %) and differentiated kidney cancer, bladder cancer, and healthy individuals (accuracy: 81.8 %). Reproduced with permission from Ref. [116].

AgNPs effectively inhibit prostate cancer (PCa) cell proliferation and induce apoptosis through multiple mechanisms, including cell cycle regulation, apoptosis induction, and inhibition of migration. In DU145 cells, AgNPs downregulate cyclin D1 and upregulate p21 and p27, resulting in G2/M phase arrest and subsequent apoptosis (Fig. 4A) [102]. Glucose-modified AgNPs (G-AgNPs) have shown selective cytotoxicity against castration-resistant prostate cancer (CRPC) cells. These nanoparticles enter cells via caveolae-mediated endocytosis, elevate reactive oxygen species (ROS), cause mitochondrial damage, and induce S-phase arrest, thereby inhibiting proliferation and triggering apoptosis [103]. Another study synthesized AgNPs via electron beam irradiation, stabilized with polyvinylpyrrolidone (PVP) and collagen hydrolysate, revealing potent anticancer activity across multiple cell lines with IC_50_ values below 1 μg/mL, indicating strong potential for selective cancer therapy [104].Green synthesis is gaining attention for AgNP preparation. For instance, PVP-coated gold, silver, and palladium nanoparticles synthesized via green reduction influenced cancer cell migration by modulating membrane lateral diffusion, while maintaining good biocompatibility [105]. Starch-coated AgNPs also exhibited significant cytotoxicity in LNCaP and PC-3 PCa cells by damaging membranes and mitochondria, inducing proliferation arrest and cell cycle blockade [106]. Additionally, a silver nanoparticle–artichoke polyphenol complex showed strong cytotoxicity in PC-3 cells by upregulating pro-apoptotic and downregulating anti-apoptotic genes, suggesting potential as an anti-PCa agent [107].

AgNPs also offer innovative platforms for highly sensitive PCa diagnostics. A peptide-templated AgNP electrochemical biosensor (CCY-LWYIKC) was developed for ultrasensitive detection of PSMA-positive exosomes by integrating exosome capture with signal amplification, significantly enhancing electrochemical responses (Fig. 4B) [108]. Another study employed an ECL-RET platform combining CeO_2_–AuNPs–g-CNQDs and AgNPs–VB_2_ to amplify and quench ECL signals, achieving PSA detection down to 0.0045 pg/mL^109^. A biomimetic SERS substrate using AgNPs embedded in PDMS, combined with electromagnetic–chemical synergy and gold nanorod immunoprobes, enabled highly sensitive PSA detection with a limit of 3.0 × 10^−10^ mg/mL [110].

Progress in green synthesis also led to a chitosan–AgNP–luminol ECL immunosensor with a PSA detection limit of 0.6 ng/mL and excellent linearity [111]. Another eco-friendly, label-free electrochemical immunosensor employed quince seed mucilage to enhance antibody immobilization, achieving a PSA detection limit of 0.078 pg/mL^112^.

#### 4.2.2 The therapy and imaging of AgNPs in bladder cancer and RCC

AgNPs exhibit significant potential in both the treatment and diagnosis of bladder cancer. Biogenic AgNPs synthesized using Fusarium sp. were shown to induce apoptosis, and inhibit migration and proliferation of 5637 bladder cancer cells. In a murine non-muscle invasive bladder cancer (NMIBC) model, they achieved 57.13 % tumor regression, with some mice showing normal urothelium or benign lesions, suggesting their potential as an alternative therapeutic approach (Fig. 4C) [113]. Another study revealed that AgNPs exert antitumor effects by inducing excessive reactive oxygen species (ROS), upregulating Bax, downregulating Bcl-2, and activating caspase-3 and -7, thereby promoting apoptosis of 5637 cells through regulation of key apoptotic pathways [114].

In diagnostics, AgNPs combined with SWATH-MS technology were employed to identify serum protein biomarkers in NMIBC patients. Significant alterations were observed in proteins associated with complement and coagulation cascades, as well as apolipoproteins, offering new candidates for early diagnosis [115]. SERS-based platforms are also widely applied to renal cancer diagnostics. Chen et al. [116] proposed a novel 3D SERS substrate integrating porous membranes and AgNPs, which significantly enhanced signal intensity and detection accuracy. Using principal component analysis–linear discriminant analysis (PCA-LDA), the classification accuracy for renal cancer reached 98.5 %, markedly surpassing that of traditional substrates (Fig. 4D).

#### 4.2.3 Applications of AgNPs in other types of urinary system tumors

In adrenal cancer diagnostics, Chen et al. [117] developed a surface-enhanced Raman spectroscopy (SERS) platform based on silver nanoparticles (AgNPs), integrated with principal component analysis and support vector machine (PCA-SVM) modeling. This approach enabled noninvasive and accurate serum-based classification of adrenal tumors, achieving classification accuracies of 96.8 % and 84.5 %, respectively.

### 4.3 Magnetic nanoparticles

Magnetic nanoparticles (MNPs), typically composed of pure metals such as iron, cobalt, or nickel, or metal-polymer composites, can be guided by external magnetic fields [118]. Their high surface area and size-dependent physicochemical properties confer superior strength, multifunctionality, and reactivity compared to bulk materials [119]. In recent years, MNPs have shown increasing utility in tumor hyperthermia, controlled drug delivery, magnetic resonance imaging (MRI), and biosensing applications (Table 5). They can modulate drug pharmacokinetics, reducing cytotoxicity and improving release rates. Targeting specificity can be further enhanced via ligand modification in addition to magnetic guidance.

**Table 5:** Therapeutic applications of MNPs in urologic tumors.

	Nanoparticle	Nanoparticle Size(nm)	Outcome	Cell Lines	Stage	Ref.
PC	FiFe@RBM	164.5	Enhancing ferroptosis via lipid metabolism reprogramming and synergize with magnetic hyperthermia to eradicate CRPC	PC3 cells	in vivo in vitro	125
	TMNPs	23.0 ± 0.8	(TMNPs) with biomimetic cell membrane and peptide modifications enhance intracellular hyperthermia.	PC3 cells	in vitro	126
	MNP-β-Glu-PEG	–	Magnetically targeted β-Glu/amygdalin therapy enhances prostate cancer inhibition.	RM1,PC3,LNCaP cells	in vivo in vitro	127
	CA-SPION	148	Developed DOX-loaded magnetic nanoparticles with strong antitumor activity in RM1 and MEC1 cells.	RM1,MEC1 cells	in vitro	128
	ZnMn-IONPs	13.32 ± 4.11	The first effective systemically delivered magnetic hyperthermia strategy using Zn/Mn-doped nanoclusters	DU145,HEK-293 cells	in vivo in vitro	129
	uSPIO-5D3-DM1-AF488/CF750	38.3	Targeted uSPIO-based theranostic nanoparticles enable multimodal imaging and selective therapy.	PC3-PIP,PC3-Flu cells	in vivo in vitro	130
	Eto-BSA@PAA@SPION	7.27	Laser-assisted SPIONs reduced IC50 to the lowest reported levels.	LNCaP,PC3,DU145 cells	in vitro	131
	Folic acid-curcumin@β-CD-MGO	210.6	A folic acid-targeted magnetic graphene oxide nanocarrier enabled efficient delivery and MRI-traceable visualization.	LNCaP,PC3 cells	in vitro	132
	CH-NP/si-BATF	83.4	Targeted siRNA delivery via chitosan nanoparticles suppressed prostate cancer by silencing BATF and PRDM1	RM-1,TRAMP-C1 cells	in vivo in vitro	133
BC	MNP-HA	30.4	A novel mild MHT strategy for precise treatment and reversal of the immunosuppressive microenvironment.	MB49 cells	in vivo in vitro	150
	MINS@MΦ	116.2 ± 12.5	Enhancing BCG immunotherapy through NIR-induced pyroptosis and M1 polarization.	RAW264.7 cells	in vivo in vitro	151
	rPAE@SPIONs	190	Enhancing bladder cancer therapy via synergistic photothermal effect, ferroptosis, and M1 polarization.	MB49 cells	in vitro	152
	Fe3O4-THP-CS/GP	–	Developed an efficient magnetic drug delivery system achieving precise in vivo SPIONs aggregation and imaging.	T24 cells	in vivo in vitro	153
	DOX-mMSs	12.8	DOX-mMSs achieve precise tumor inhibition via magnetic retention and pH-triggered release.	NIH/3T3 cells	in vivo in vitro	154
	Fe/DOX@SL	100	Fe/DOX@SL microrobots enable precise propulsion and efficient bladder cancer suppression via RMF guidance.	T24 cells	in vivo in vitro	155
	293T-R-Fe@OA	15.45 ± 1.78 μm	Integrating tumor-specific targeting and virotherapy for precise and efficient bladder cancer inhibition.	T24 cells,293T cells	in vivo in vitro	156
Others	FA-DEX-SPION	74 ± 12	FA-DEX-SPION targeted VNC delivery induces cancer cell apoptosis	Tera-1,Hs1 cells	in vitro	160

MNPs also serve as effective MRI contrast agents by enhancing proton relaxation effects at targeted tissues, thereby improving image clarity [120]. For instance, ultrasmall MnFe_2_O_4_ nanoparticles with high saturation magnetization have been developed for advanced MRI applications [121]. Superparamagnetic iron oxide nanoparticles (SPIONs) have gained widespread attention in magnetic particle imaging (MPI) due to their high sensitivity, enabling clear visualization of vasculature, organs, and tumor metastases [122,123]. Beyond imaging, SPIONs exhibit strong magnetic heating capabilities, offering advantages over photothermal therapy by effectively targeting deep-seated tumors [124] (Table 6).

**Table 6:** Diagnostic and theranostic applications of MNPs in urologic tumors.

	Nanoparticle	Nanoparticle Size(nm)	Outcome	Cell Lines	Stage	Ref.
PC	MNP@template	350	MIP-M offering a stable and specific alternative to antibodies for prostate cancer imaging.	LNCaP, PC3,22Rv1 cells	in vivo in vitro	134
	Raman beads	300	microfluidic Raman biochip for rapid exosome analysis, achieving a detection limit of 1.6 × 10^2^ particles/mL	LNCaP, PrEC cells	in vitro	135
	MxFe3−xO4	6	Elucidation of MRI contrast enhancement mechanism of transition-metal-doped IONs, optimization of Zn-doped IONs	–	in vivo	136
	Fe3O4@SiO2@TiO2	600	Developed a rapid and efficient exosome detection platform using TiO_2_ capture and PSMA aptamer recognition.	–	in vitro	137
	68Ga-mNP-N1/2	73.6	Developed dual-targeted 68Ga-mNPs for efficient PET/MRI imaging of prostate cancer via PSMA and GRPR.	PC-3,LNCaP cells	in vitro	138
	H2N-Fe_3_O_4_	7.4	H2N-Fe_3_O_4_ nanoparticles enabled significant early-phase T1/T2 MRI contrast enhancement in prostate tumors in vivo.	–	in vivo	139
	PSN NPs	23 ± 6	A nerve-targeted MRI/MPI nanoprobe enables precise in vivo visualization by delivering β-blockers for tumor suppression.	DU-145luc,PC-3luc cells	in vivo in vitro	140
	Fe3O4@DPA-PEG-PSMA-1	35.1 ± 11.6	Established a Glu-Urea-Lys-based PSMA-targeted SPION probe for sensitive MRI diagnosis of prostate cancer in vivo.	LNCaP,PC3,HaCaT cells	in vivo in vitro	141
	USPIO(Cy7.5)-BBN	4.93 ± 0.31	USPIO(Cy7.5)-BBN nanoparticles show high specificity and efficacy in targeting GRPr-expressing prostate cancer cells,.	PC-3 cells	in vivo in vitro	142
	SPION/CCh/N-cad	223	SPION/CCh/N-cad enables rapid and specific magnetic capture of prostate cancer circulating tumor cells.	PC-3,DU 145 cells	in vitro	143
	C-Fe3O4 NPs	38–50	Designed a PSMA-targeted SPION theranostic probe enabling PET/MRI imaging and radionuclide therapy.	LNCaP,PC3 cells	in vitro	147
	ICG-SPION	3.5–10	The first demonstration of the feasibility of radiation-free sentinel lymph node dissection (sLND).	–	in vivo	148
	SPIONs@EXO-dye	63.6	(SPIONs@EXO-Dye) enabled targeted imaging and combined photothermal–magnetothermal therapy	PC3 cells	in vivo in vitro	149
BC	SNSC-MNPs	180–240	Developed a recyclable SNSC-MNPs cascade for efficient DNA digestion and 5hmC detection within 30 min.	T24 cells	in vitro	157
	177Lu-Fe3O4@HA/DBCO	7.0	A novel 177Lu-labeled Fe_3_O_4_ nanoprobe enabled precise MRI imaging and internal radiotherapy for bladder cancer,	T24 cells	in vivo in vitro	158
	FePPy-NH2 NPs	54.3	FePPy-NH2 NPs achieve efficient photothermal therapy guided by dual-mode MRI and PAI imaging.	Ealy926 cells	in vivo in vitro	159
Others	MMIP	–	Magnetic MIP-based d-SPE enables rapid enrichment and sensitive detection of urinary PPGL biomarkers.	–	in vitro	162

#### 4.3.1 Precision delivery and magnetic hyperthermia in prostate cancer

**Fig. 5:**
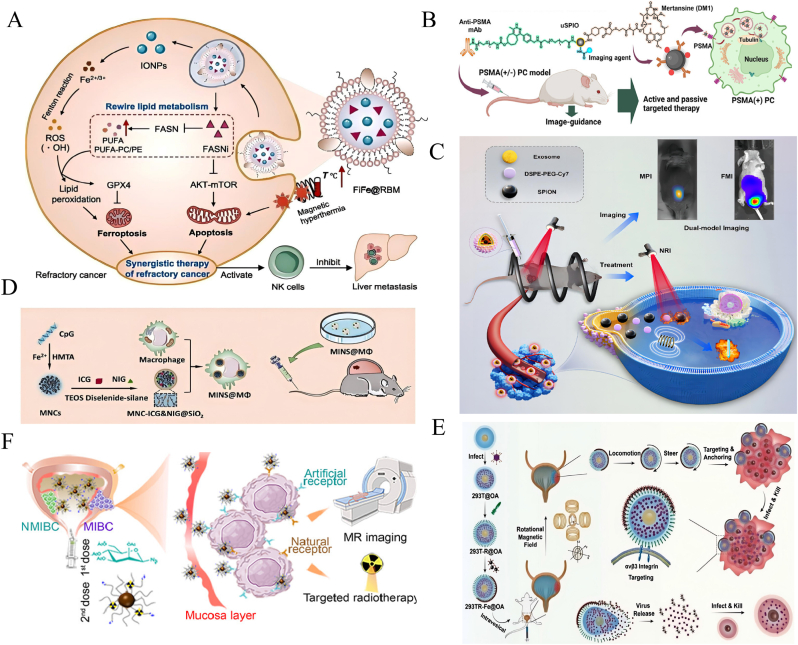
**Application of MNPs in urinary system tumors. A:** Schematic illustration of engineering FiFe@RBM and the mechanism of rewiring lipid metabolism to enhance ferroptosis and synergize with magnetic hyperthermia for effective treatment of CRPC and inhibiting liver metastasis through activated NK cells. Reproduced with permission from Ref. [125].**B:** Design of uSPIO-5D3-DM1-AF488/CF750 nanoparticles targeting PSMA for image-guided active and passive therapy in prostate cancer (PC). Confocal microscopy confirms selective uptake in PSMA(+) cells, and tumor volume curves indicate significant growth inhibition in PSMA(+) models treated with targeted nanoparticles. Reproduced with permission from ref.^130.^**C:** Metabolic imaging and thermotherapy using patient-derived exosome-loaded nanoplatform (SED) in orthotopic and subcutaneous prostate cancer models, facilitated by dual-modality imaging. Reproduced with permission from Ref. [149].**D:** Preparation and mechanism of action of the engineered macrophages. Reproduced with permission from Ref. [151].**E:** Schematic of magnetic-driven OA-loaded 293T cell robots actively delivering targeted virotherapy for bladder cancer. Modified with cRGD and Fe_3_O_4_ nanoparticles, these robots exhibit directional migration, enhanced tumor targeting, and effective virus-mediated cytolysis. Reproduced with permission from Ref. [156].**F:** Schematic Drawing to Show the Theranostic Agent, *i.e.*, 177Lu-Fe_3_O_4_@HA/DBCO, for MRI Imaging and Targeted Radionuclide Therapy of Bladder Cancer. Reproduced with permission from Ref. [158].

Magnetic nanoparticles (MNPs) have shown significant advances in the treatment of prostate cancer, with iron oxide-based MNPs being extensively studied. Cheng et al. [125] designed biomimetic FiFe@RBM nanovesicles that integrate lipid metabolism reprogramming with magnetic hyperthermia (MHT) (Fig. 5A). In PC-3 models, these vesicles induce ROS generation and mitochondrial damage, activate NK cells, and synergistically trigger apoptosis and ferroptosis, thereby effectively inhibiting tumor metastasis. Trimagnetic nanoparticles (TMNPs), engineered with a core–shell structure and dual-modified by prostate cancer cell membranes and cell-penetrating peptides, suppress PCa cell proliferation and migration under alternating magnetic fields (AMF) via a caspase-9-mediated apoptotic pathway [126]. Starch-coated MNPs combined with amygdalin enhance antitumor effects through an enzyme–prodrug strategy while exhibiting good biocompatibility [127]. In another study, multifunctional iron oxide MNPs modified with folic acid showed improved targeting of prostate cancer cells and efficient doxorubicin (DOX) delivery and release [128]. Additionally, Zn/Mn co-doped hexagonal ferrite nanoparticles encapsulated in polymers demonstrated potent thermal ablation and tumor suppression under magnetic heating [129]. Superparamagnetic iron oxide nanoparticles (SPIONs) also exhibited therapeutic potential. A uSPIO theranostic agent conjugated with a PSMA antibody and DM1 microtubule inhibitor enabled dual-modal MRI and NIR imaging, effectively suppressing PSMA-positive tumors (Fig. 5B) [130]. Another approach used bovine serum albumin-coated SPIONs for etoposide (Eto) delivery combined with photothermal therapy, significantly enhancing PCa cell killing under NIR irradiation with high biocompatibility [131].Other MNP-based systems have also made progress. A folate-targeted Cur@β-CD-MGO system significantly improved curcumin bioavailability, enabling controlled release and targeted cytotoxicity against prostate cancer cells [132]. Chitosan-coated MNPs delivering siRNA against BATF and PRDM1 in Treg cells attenuated immunosuppression, thereby inhibiting tumor growth and metastasis [133].

Zhang et al. [134] developed PSMA-imprinted nanogels (MIP-M) with specificity comparable to antibodies while avoiding their instability and toxicity, offering a new platform for precise imaging and therapy. A microfluidic Raman biochip combining anti-CD63 MNPs and EpCAM-functionalized Raman beads efficiently captured and analyzed exosomes from clinical PCa serum samples [135]. For MRI applications, transition metal-doped iron oxide nanocrystals (IONs) enhanced T_2_ contrast and demonstrated safe and efficient imaging in PCa mouse models [136]. Another dual-functional probe combining Fe_3_O_4_@SiO_2_@TiO_2_ particles and PSMA aptamers enabled rapid exosome detection via reversible “on–off” signal modulation, outperforming traditional NTA methods [137]. A study utilizing ^68^Ga-labeled iron oxide nanoparticles conjugated with dual-targeting ligands for PSMA and GRPR revealed excellent imaging performance in PET/MRI for distinct PCa phenotypes [138]. Amino-functionalized Fe_3_O_4_ (H_2_N-Fe_3_O_4_) also showed strong tumor accumulation and MRI potential in early PCa detection [139].

Notable breakthroughs have been achieved in SPION-based imaging. Propranolol–neuropeptide-conjugated SPIONs (PSN NPs) enabled highly sensitive visualization of perineural invasion in PCa via MRI and MPI [140]. PSMA-targeted SPIONs functionalized with Glu–Urea–Lys scaffolds demonstrated excellent physicochemical stability and specificity, allowing accurate molecular MRI in mouse models [141]. A bimodal MRI/fluorescence nanoprobe based on ultrasmall SPIONs conjugated with Cy7.5-labeled BBN [7–14] peptide targeted GRPR with high affinity and signal strength [142]. The SPION/CCh/N-cad system, modified with chitosan derivatives and anti-N-cadherin antibodies, efficiently captured circulating tumor cells (CTCs) in a short time, confirming its potential for PCa monitoring and diagnosis [143].

Recent advances have demonstrated that nanoparticle-based imaging probes and chemoimmunotherapy strategies can visualize PD-L1 expression, remodel the tumor immune microenvironment, and enhance synergistic radioimmunotherapy [[144], [145], [146]]. MNPs have demonstrated excellent performance in integrated imaging and therapy for prostate cancer. A SPION-based radiotheranostic system combined PSMA-617 targeting with ^44^Sc/^47^Sc chelation, enabling PET/MRI imaging and radiotherapy, and achieved significant therapeutic efficacy in PSMA-positive LNCaP cells and 3D tumor spheroids [147]. A SPION–indocyanine green composite probe allowed radiation-free sentinel lymph node mapping and metastasis detection during surgery [148]. The SPIONs@EXO-Dye platform, built from patient-derived exosomes, offered dual-modality MPI/FMI imaging along with targeted metabolic tracking and photothermal/magnetic hyperthermia therapy (Fig. 5C) [149].

#### 4.3.2 Image-guided drug delivery for bladder cancer using MNPs

Magnetic nanoparticles (MNPs) demonstrate significant potential in bladder cancer therapy. Mild magnetic hyperthermia (MHT), employing intravesical instillation of hyaluronic acid-modified MNPs, enhances the targeting of CD44 and RHAMM receptors on bladder cancer cells, reduces HSP70, cyclin D1, and Bcl-2 expression, promotes tumor apoptosis, and generates sustained adaptive immune responses [150]. Additionally, engineered macrophages (MINS@MΦ), guided by BCG-induced local inflammation, accumulate specifically at tumor sites; near-infrared laser irradiation induces macrophage autophagy, releasing Fe^2+^ and CpG, thereby enhancing M1 polarization and secretion of antitumor cytokines (Fig. 5D) [151]. A pomegranate-like nanoplatform based on superparamagnetic iron oxide nanoparticles (SPIONs) significantly enhances chemotherapy efficacy via photothermal effects and iron overload-induced ferroptosis, further augmented by M1 macrophage polarization [152]. Sun et al. [153] developed covalently linked SPION–drug conjugates, achieving rapid and precise drug delivery under magnetic guidance, and monitored drug distribution using a custom magnetic particle imaging (MPI) system. Doxorubicin-loaded magnetic hydrogel microspheres (DOX-mMSs), composed of chitosan (CS) and polyvinyl alcohol (PVA), enable magnetically controlled retention and precise drug release regulated by urinary pH, increasing bladder retention time and antitumor effects [154]. Micro-robot technology employing Fe_3_O_4_ nanoparticles combined with spores of the traditional Chinese herb SL and doxorubicin (Fe/DOX@SL) allows precise drug delivery guided by rotating magnetic fields, achieving notable therapeutic outcomes [155]. Additionally, a cell-based microrobot platform (293T-R-Fe@OA), integrating oncolytic adenovirus and Fe_3_O_4_ nanoparticles, significantly improves virus retention and tissue penetration at tumor sites through magnetic control, enabling precise and effective viral therapy (Fig. 5E) [156].

For bladder cancer diagnosis and theranostics, magnetic nanoparticles offer remarkable advantages in multimodal imaging and multifunctional therapies. The SNSC-MNPs system, integrating snake venom phosphodiesterase (SVP), calf intestinal alkaline phosphatase (CIP), and supernuclease (SN) onto MNPs, rapidly digests DNA within bladder cancer cells, accurately measuring changes in 5-hydroxymethylcytosine (5hmC) induced by tetrachlorobenzoquinone (TCBQ) via HPLC-MS/MS within 30 min [157]. A hyaluronic acid-coated multifunctional nanoprobe incorporating dibenzocyclooctyne (DBCO) provides targeted MRI imaging of bladder cancer and effective stage reduction and metastasis inhibition through internal radiotherapy with ^177^Lu (Fig. 5F) [158]. Moreover, Fe(III)-doped polypyrrole nanoparticles (FePPy-NH_2_ NPs) combining MRI, photoacoustic imaging (PAI), and photothermal therapy (PTT) enable precise tumor imaging and efficient elimination, demonstrating high tumor accumulation and excellent biosafety [159].

#### 4.3.3 Applications of MNPs in other types of urinary system tumors

The application of magnetic nanoparticles (MNPs) continues to expand in other urological cancers. In testicular cancer, a novel FA-DEX-SPION nanocarrier delivering vincristine (VNC) specifically targets Tera-1 cells through folate mediation, achieving pH-responsive release, over tenfold increased cytotoxicity, and effective apoptosis induction via modulation of Caspase-9, P53, P21, and Akt1 pathways [160]. In diagnostic imaging, lymphotropic nanoparticle-enhanced MRI (LNMRI) using Ferumoxtran-10 demonstrated 100 % sensitivity, specificity, and negative predictive value for detecting regional lymph node metastasis in penile cancer patients, significantly aiding surgical decision-making [161]. Additionally, Zeng et al. [162] developed a magnetic molecularly imprinted polymer (MMIP)-based dispersive solid-phase extraction (d-SPE) system, efficiently isolating pheochromocytoma metabolites from urine. Combined with HPLC-FLD/UVD, it achieved an AUC of 0.975, demonstrating robust potential for liquid biopsy diagnostics.

### 4.4 Mesoporous silica nanoparticles

Mesoporous silica nanoparticles (MSNs), inorganic nanomaterials with ordered pore structures, adjustable pore sizes (typically 2–10 nm), and large surface areas, exhibit broad biomedical applications, particularly in targeted tumor therapy and drug delivery systems [163] (Table 7). Their porous structure ensures excellent drug loading and solubility, enabling encapsulation and controlled release of macromolecules, such as immunoproteins [164,165]. Additionally, hollow MSNs with increased internal space and lower density are ideal carriers for enzyme delivery [166,167]. MSNs also integrate nanovalve technologies responsive to internal or external stimuli (e.g., pH, redox conditions, enzymes, temperature, magnetic fields, light, and ultrasound), enabling precise drug release [168]. These stimuli-responsive MSNs provide smart therapeutic strategies with selective drug release at specific disease sites, enhancing therapeutic efficacy and reducing side effects, demonstrating significant potential in diagnosis and treatment of urological cancers (Table 8).

**Table 7:** Therapeutic applications of MSNs in urologic tumors.

	Nanoparticle	Nanoparticle Size(nm)	Outcome	Cell Lines	Stage	Ref.
PC	CSMS	140–600	Hexagonal prism-shaped CSMS shows superior uptake and anticancer efficacy over spherical CSMS in PC3 cells.	PC3 cells	in vivo in vitro	169
	nanoPMOs	181 ± 29	Quantified degradation and targeting efficiency of antibody-conjugated nanoPMOs using dSTORM imaging.	LNCaP,RWPE-1 cells	in vitro	170
	TE-@MS-11	440–490	Single-step synthesis of egg-yolk core-shell MSNs achieved a high Dox loading efficiency of 481 μg mg^−1^.	PC-3、LNCaP and RWPE-1	in vitro	171
	TBZ MCM-41	215.9 ± 0.07	Enhancing the anticancer activity of thiabendazole in prostate cancer cells by improving solubility and ROS generation.	PC-3 cells	in vitro	172
	MCM-BLG	250 ± 2	Enhancing FBZ solubility, show pH-dependent release, and increase cytotoxicity and ROS generation in PC-3 cells.	HEK-293,PC-3 cells	in vitro	173
	HNG-P(DEGMA)	206.9	Hybrid nanogels exhibit dual pH and thermo-responsiveness, controlled drug release, and significant anticancer efficacy.	NIH 3T3,LNCaP cells	in vitro	174
	PEG-MCM-FBZ	366.3 ± 6.9	Enhancing FBZ solubility and cytotoxicity against PC-3 cells, showing 3.8-fold increased anticancer effect.	PC-3 cells	in vitro	175
BC	THP@CHPS NPs	326.1 ± 14.8	CPBA-modified nanosystem enhances drug targeting and retention in bladder cancer cells.	NIH 3T3,MB49 cells	in vivo in vitro	177
	MSN-SH(E)	90–120	MSN-SH(E) with MMC improves adhesion, permeation, immunity, enhancing NMIBC treatment.	J774a.1 cells	in vivo in vitro	178
	CREKA@LPT-MSNC	180	CREKA@LPT-MSNC inhibits platelet function and enhances cisplatin delivery, improving bladder cancer treatment.	MB49 cells	in vivo in vitro	179
	PLACS NPs	140	PLACS NPs enable precise multiplex gene activation, enhancing anti-tumor effects in bladder cancer.	5637,T24,SV-HUC-1 cells	in vivo in vitro	180
	DOX/MSN/Chimera	150	DOX/MSN/Chimera effectively targets resistant bladder cancer, reducing proliferation and enhancing apoptosis.	SV-HUC-1,T24 cells	in vivo in vitro	181
	MSNP-100,	117 ± 16	MSNP-based nanocarriers enhance photosensitizer delivery and photodynamic therapy in bladder cancer.	HT-1376,UM-UC-3 cells	in vitro	182
	MSNs	60–160	Core-shell MSNs achieved the highest gene knock-down and antitumoral efficacy through rapid EGFR-targeted cellular internalization.	T24 cells	in vitro	183
	N23BP-CodA-SiNP	17	Smaller 17 nm nanoparticles showed enhanced distribution and retention in tumor spheroids.	HTB9 cells	in vitro	184
RCC	MLP@M	211.1 ± 9.4	MLP@M enhanced RCC suppression with precise targeting, prolonged retention, and effective tumor inhibition.	786-O cells	in vivo in vitro	185

**Table 8:** Diagnostic and theranostic applications of MSNs in urologic tumors.

	Nanoparticle	Nanoparticle Size(nm)	Outcome	Cell Lines	Stage	Ref.
PC	PSA-Mn-Msn-Cy7	46–52	Achieved high T1 relaxivity, precise PSA-targeted imaging, and dual-modal detection of prostate cancer.	LNcap cells	in vivo in vitro	176
Others	EB@HMSN(E)-SH	192 ± 29	EB@HMSN(E)-SH improves tumor detection via enhanced mucoadhesion and permeation under WLC.	MBT-2, NBT-2 cells	in vivo in vitro	186
	WSNs	140 ± 5	WSNs-based fluorescence assay enables multiplex miRNA detection with high sensitivity and specificity.	–	in vitro	187

#### 4.4.1 MSN-based Co-delivery systems for prostate cancer

**Fig. 6:**
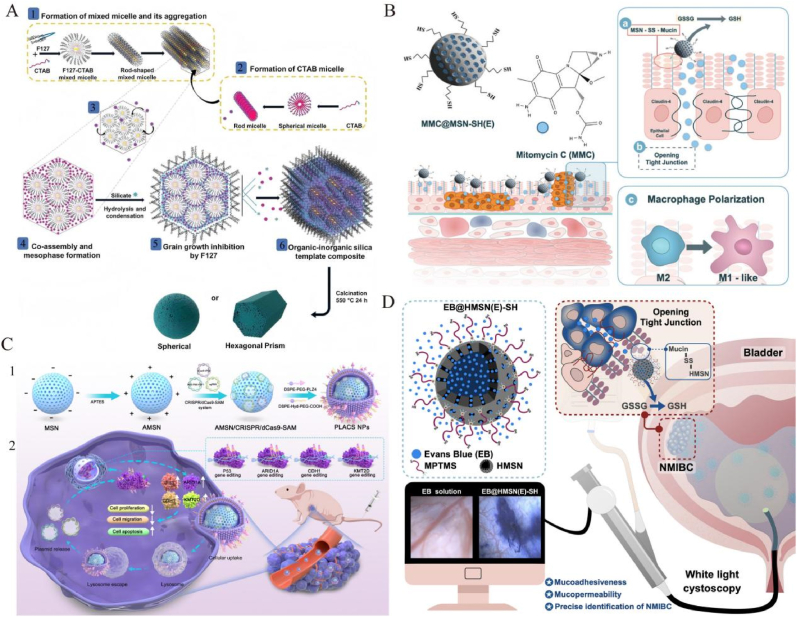
**Targeted delivery using MSNs in urinary system tumors. A:**Schematic of synthesizing prismatic hexagonal mesoporous silica nanoparticles via mixed micelle (F127/CTAB) formation, mesophase assembly, silicate condensation, and controlled calcination, yielding either spherical or hexagonal prism shapes depending on solvent conditions. Reproduced with permission from Ref. [169].**B:**Schematic diagram of MMC@MSN-SH(E) nanoformulation for the treatment of non-muscle invasive bladder cancer. Possible therapeutic mechanisms include a) mucoadhesion, b) permeation enhancement, and c) macrophage polarization. Reproduced with permission from Ref. [178].**C:**Preparation and in vivo delivery of CRISPR/dCas9-SAM-loaded mesoporous silica nanoparticles (PLACS NPs) for multiplex activation of tumor suppressors (P53, ARID1A, CDH1, KMT2D), promoting apoptosis and inhibiting bladder cancer progression. Reproduced with permission from Ref. [180].**D:** Schematic diagram illustrating the application of EB@HMSN(E)-SH for bladder cancer detection. EB@HMSN(E)-SH was introduced into the bladder cavity via the urethra. Facilitated by the EB@HMSN(E)-SH particles, EB was precisely targeted to the tumor tissue, allowing experimental visualization of the tumor using WL cystoscopy. Reproduced with permission from Ref. [186].

Mesoporous silica nanoparticles (MSNs) exhibit significant advantages in prostate cancer diagnosis and therapy, including efficient drug delivery, controlled release, excellent targeting ability, and enhanced anticancer efficacy, making them promising candidates for precision medicine. Mohanan et al. [169] synthesized core-shell mesoporous silica nanoparticles (CSMS) via a dual-surfactant-assisted method for loading cabazitaxel (CBZ), demonstrating high cellular uptake and enhanced anticancer activity. Particularly, hexagonal prism-shaped CSMS displayed superior drug release efficiency and cytotoxicity (Fig. 6A). Das et al. [170] developed antibody-conjugated biodegradable silica nanoparticles (nanoPMOs) characterized by dSTORM imaging, which efficiently delivered doxorubicin to prostate cancer cells, significantly enhancing targeted anticancer effects. Nitrogen-rich, surface-functionalized MSNs exhibited high doxorubicin loading capacity, increased cytotoxicity, and improved cellular uptake efficiency, highlighting their potential in prostate cancer chemotherapy [171]. Another study loaded thiabendazole (TBZ) onto MCM-41 MSNs, enhancing drug solubility and anticancer activity, achieving controlled release and increased cytotoxicity under acidic conditions [172]. Furthermore, MCM-48 nanoparticles modified with β-lactoglobulin (BLG) and loaded with fenbendazole (FBZ) showed significantly enhanced cytotoxicity and pH-dependent drug release in PC-3 cells [173]. Hybrid nanogels (HNGs) synthesized via ultrasound-assisted radical polymerization exhibited excellent controlled-release profiles for camptothecin (CPT), demonstrating potent inhibitory effects on prostate cancer cells, along with favorable biocompatibility and stimulus-responsive behavior [174]. PEGylated MCM-41 nanoparticles loaded with fenbendazole (PEG-MCM-FBZ) significantly enhanced cytotoxicity and ROS generation, representing an effective vehicle to improve drug solubility and anticancer efficacy [175].

In prostate cancer diagnostics, Du et al. [176] designed prostate-specific antigen (PSA)-targeted manganese-doped mesoporous silica nanoparticles (PSA-Mn-MSN-Cy7) for dual-modal fluorescence and MRI imaging. In vitro and in vivo studies showed selective accumulation of PSA-Mn-MSN-Cy7 in prostate cancer cells, resulting in strong optical and T1 signals, without significant aggregation in non-cancerous cells.

#### 4.4.2 Mucoadhesive MSNs in bladder cancer and RCC: localized and controlled release

Mesoporous silica nanoparticles (MSNs) exhibit considerable potential in bladder and renal cancer treatments. In bladder cancer therapy, Wang et al. [177] developed hollow pollen-like silica nanoparticles (HPS NPs) modified with CPBA, enhancing targeted binding and retention, resulting in significant antitumor effects. Similarly, thiolated hollow MSNs (MSN-SH(E)) improved mucoadhesion and reprogrammed macrophages from an M2 to M1 phenotype, augmenting immunotherapeutic efficacy (Fig. 6B) [178]. Another system, CREKA-functionalized nanoparticles (CREKA@LPT-MSNC), combining cisplatin and antiplatelet drug tirofiban, effectively inhibited lymphovascular invasion and metastasis [179]. Xu et al. [180] employed PLACS NPs carrying the CRISPR/dCas9-SAM system, activating multiple tumor suppressor genes, significantly reducing proliferation and migration of bladder cancer cells (Fig. 6C). The DOX/MSN/Chimera system, integrating anticancer drugs with siRNA chimeras, targeted resistant bladder cancer cells and demonstrated effective controlled drug release and tumor inhibition [181]. In photodynamic therapy (PDT), MSNP carriers (spherical vs. rod-shaped) improved photosensitizer uptake and phototoxicity, with spherical MSNP-PS2 notably enhancing internalization and efficacy against HT-1376 and UM-UC-3 cells [182]. Furthermore, nanoparticle size critically influences targeting efficiency; 160 nm APTES-modified MSNs rapidly delivered antitumor microRNA (miRNA), achieving effective gene knockdown and tumor suppression [183]. Likewise, smaller 17 nm silica nanoparticles with targeting ligands demonstrated enhanced tumor penetration and accumulation [184]. For renal cancer, Chen et al. [185] developed a laser-responsive MSN system (MLP@M) combining heat-sensitive mitochondrial metabolism inhibitors and photothermal materials, significantly inhibiting tumor growth under 808 nm irradiation in mouse models.

For early bladder cancer diagnostics, Fa et al. [186] introduced EB@HMSN(E)-SH nanoparticles, combining external thiolation and Evans Blue dye loading, enhancing dye penetration and retention in tumors (Fig. 6D). This platform, in conjunction with conventional white-light cystoscopy (WLC), effectively distinguished tumors from normal or inflammatory tissues, particularly small, flat lesions, thereby significantly improving diagnostic accuracy. Additionally, another study utilized wrinkled-structure silica nanoparticles (WSNs) to construct an enzyme-free, amplification-free fluorescent microreactor for multiplexed miRNA detection. Employing S9.6 antibodies for miRNA enrichment and recognition, the system achieved ultrasensitive detection (LOD = 5 fM) and single-base mismatch discrimination across a dynamic range spanning six orders of magnitude, demonstrating robust stability and accuracy in clinical serum samples and presenting a powerful strategy for noninvasive bladder cancer screening and personalized medicine [187].

## 5 Organic nanoparticles

### 5.1 Liposomes

Liposomes, composed of phospholipid bilayers, encapsulate both hydrophilic and hydrophobic drugs and exhibit favorable biodegradability and low toxicity, making them widely used drug-delivery vehicles [188]. They leverage the enhanced permeability and retention (EPR) effect to achieve passive targeting, thus enhancing drug bioavailability and selective delivery, reducing normal tissue toxicity, and prolonging drug retention time in vivo [189,190]. As the first FDA-approved nanoplatform for cancer therapy, liposomes have shown particular promise in treating urological cancers [191] (Table 9). The recent success of lipid nanoparticles (LNPs) in COVID-19 mRNA vaccines has renewed interest in their potential as nucleic acid carriers [192,193]. Current research emphasizes enhancing liposomal stability and targeting capabilities, such as using ligands like RGD peptides to improve tumor localization and cellular uptake, further advancing their therapeutic potential and safety [194].

**Table 9:** Therapeutic applications of liposomes in urologic tumors.

	Nanoparticle	Nanoparticle Size(nm)	Outcome	Cell Lines	Stage	Ref.
PC	Dox-PSA-loaded LTSL	139	Thermosensitive liposomes encapsulate Dox-PSA, enhancing prostate cancer therapy and minimizing systemic toxicity.	C4-2B,LNCaP,PC3 cells	in vivo in vitro	195
	Sim-Dox liposomes	174	Liposomal co-encapsulation of simvastatin and doxorubicin reduces cardiotoxicity while enhancing the anti-cancer effect.	PC3,H9c2 cells	in vivo in vitro	196
	LTSL-BPNF-Dox	135.8 ± 2.01	Developed temperature-sensitive liposomes co-loaded with black phosphorus nanoflakes and docetaxel.	PC-3,DU 145 cells	in vitro	197
	Doc/Res-LPs	99.76 ± 3.14	Doc/Res co-loaded liposomes enhance synergistic antitumor efficacy and reduce toxicity for prostate cancer therapy.	PC3,DU145 cells	in vitro	198
	Cis-Dox-TSL	120 ± 10	Combination of cisplatin and doxorubicin in temperature-sensitive liposomes enhances antitumor effects.	LNcaP cells	in vivo in vitro	199
	CUR + pEGCG/POPC	100	CUR/pEGCG co-loaded liposomes enhance anticancer activity and polyphenol stability for urogenital cancer therapy.	5637,MRC-5, LNCaP cells	in vitro	200
	LP-BC	–	Liposomal β-carotene inhibits prostate cancer proliferation and EMT, outperforming free β-carotene.	PC-3,Du145,LNCaP cells	in vitro	201
	GPL∗	60	PSMA-targeted liposomes with genistein and plumbagin inhibit prostate cancer cell proliferation and induce apoptosis.	PC-3,LNCaP cells	in vitro	202
	lipo SA-CTX	120–140	A facile and universal method was constructed to achieve liposomal remote loading of non-ionizable drugs.	RM-1 cells	in vivo in vitro	203
	iRGD-liposome-AS0 1	150 ± 36	iRGD-functionalized liposomes enhance AR-ASO delivery and efficacy in prostate cancer and bone metastasis.	22Rv1, LNCaP, VCaP cells	in vivo in vitro	204
	Gos/cRGD-LP	61.86 ± 1.73	cRGD-liposomes enhance AT-101 tumor targeting and antitumor efficacy with improved safety in vivo.	PC-3 cells	in vivo in vitro	205
	Cu(TPZ)2 liposomes	160–180	Cu(TPZ)_2_ liposomes enhance drug solubility, sensitizing prostate cancer spheroids effectively to radiotherapy.	C4-2B cells	in vitro	206
	CBZ immunoliposome	–	Anti-EGFR immunoliposomes loaded with cabazitaxel significantly enhanced cytotoxicity and therapeutic efficacy.	DU145,PC3 cells	in vitro	207
	Arsonoliposome	125	Developed ether-linked arsonoliposomes with comparable anticancer activity and improved stability.	PC3,LLC cells	in vitro	208
	pLipo	153.9	PSA-driven perforin-liposomes enhance immune cytotoxicity and inhibit chemoresistant prostate cancer growth effectively.	22Rv1DR,PC3 cells	in vitro	209
	NLPs-RGD-Cur-ATO	100	RGD-modified nanoliposomes for co-delivery of arsenic trioxide and curcumin enhance antitumor efficacy.	PC-3 cells	in vitro	210
BC	LRO-BCG/CS	110	Co-delivery of oxaliplatin prodrug liposomes and low-dose BCG enhances chemo-immunotherapy efficacy.	MB49 cells	in vivo in vitro	213
	Lipo-ELE/Ce6	158.53 ± 1.33	A ROS-responsive Ce6/elemene co-loaded liposome was developed to enhance NMIBC apoptosis via photo-triggered synergistic release.	T24 cells	in vivo in vitro	214
	ATF24-PEG-Lipo-β-E	79.32 ± 1.282	ATF24-targeted liposomes co-delivering β-elemene and cisplatin significantly enhance cancer treatment efficacy.	RT-4, KU-19-19 cells	in vivo in vitro	215
	PEG-Lipo-β-E	83.31 ± 0.181	PEGylated β-E liposomes enhance β-E's bioavailability and anticancer effects, improving treatment outcomes.	T24,KU-19-19 cells	in vivo in vitro	216
	EphA2-ILs-DTXp	113.6	EphA2-targeted nanotherapeutics enhance docetaxel efficacy, showing promising preclinical results in PDX models.	–	in vivo in vitro	217
	LCispt, PLCispt	221–274	PEGylated liposomes boost cisplatin efficacy and reduce toxicity for targeted bladder cancer therapy.	HTB-9 cells	in vivo in vitro	218
	LipoEPI:CPX	83.2–97.0	Liposomal ciprofloxacin enhances anticancer efficacy and abolishes epirubicin cardiotoxicity.	T24 cells	in vivo in vitro	219
	Lip-TDM	100–160	Cationic liposome TDM induces antitumor immunity by activating CD8^+^ T cells and dendritic cells.	B16F10,MB49,MC38 cells	in vitro	220
	CWS-Nano-CL-chitosan	196	Liposome BCG-CWS suppresses bladder cancer via AMPK-mediated ROS and ER stress, safely and effectively.	5637 cells,HT1376 cells	in vitro	221
	3025@ML	100–200	3025@ML boosts oxaliplatin efficacy via metabolic inhibition and apoptosis enhancement in bladder cancer cells.	T24,5637 cells	in vivo in vitro	222
RCC	lipo-sunitinib	173.4	PEG-liposome-delivered sunitinib enhances autophagy, immunity, and safety for targeted renal cancer therapy.	RENCA cell, A498 cells	in vivo in vitro	223
	Lipo@Suni	197.2	Renal arterial Lipo@Suni boosts tumor retention and efficacy while reducing systemic toxicity in RCC therapy.	Renca-luc cells	in vivo in vitro	224
	RES-lips	102.1 ± 10.5	Resveratrol liposomes reverse sorafenib resistance via PI3K-AKT-mTOR and VHL-HIF dual-pathway modulation.	786-O/S cells	in vivo in vitro	225
	Axi/siRNAPD−L1@NGR-Lipo	156.2	Targeted liposomes co-delivering Axi and PD-L1 siRNA normalize vasculature and boost antitumor immunity in RCC.	Renca, HUVEC cells	in vivo in vitro	226
	EY-L	67.15 ± 0.31	Tumor-targeted EY-L sensitizes RCC to radiation by inhibiting DNA repair and inducing mitotic catastrophe.	786-O cell	in vivo in vitro	227
Others	Lip-Epi + TH1-5	108.1 ± 4.67	Hepcidin liposomes synergize with Epi to reverse tumor MDR	SCC15,NT2D1 cells	in vitro	228

#### 5.1.1 Intravesical liposomal formulations for enhanced PCa therapy

In prostate cancer (PCa) therapy, liposomal drug-delivery systems have demonstrated considerable potential. For instance, Pereira et al. [195] developed low-temperature-sensitive liposomes (LTSLs) encapsulating Dox-PSA, a prodrug activated selectively by prostate-specific antigen (PSA) under mild hyperthermia, significantly improving drug selectivity and minimizing systemic toxicity. Another study co-encapsulated docetaxel (Dox) and simvastatin (Sim) in liposomes, effectively reducing Dox-associated cardiotoxicity while enhancing antitumor efficacy [196]. Additionally, combining temperature-sensitive liposomes with phototherapy, black phosphorus nanosheets (BPNFs) loaded with Dox achieved controlled release under near-infrared (NIR) irradiation, significantly improving anticancer activity [197]. PEGylated liposomes carrying docetaxel (Doc) and resveratrol (Res) significantly improved therapeutic synergy and prolonged survival in mouse models [198]. Moreover, cisplatin (Cis) and Dox encapsulated in temperature-sensitive liposomes (TSLs) combined with mild hyperthermia significantly inhibited tumor growth and promoted apoptosis, suggesting a novel combination liposomal therapy [199]. Co-loaded liposomes containing curcumin (CUR) and acetylated epigallocatechin gallate (pEGCG) markedly improved storage stability and anticancer activity of these unstable polyphenolic compounds [200]. Liposomal β-carotene (LP-BC) not only inhibited tumor cell proliferation but also effectively suppressed epithelial-mesenchymal transition (EMT) without activating it, potentially acting via modulation of androgen-related pathways and lipid metabolism [201].

**Fig. 7:**
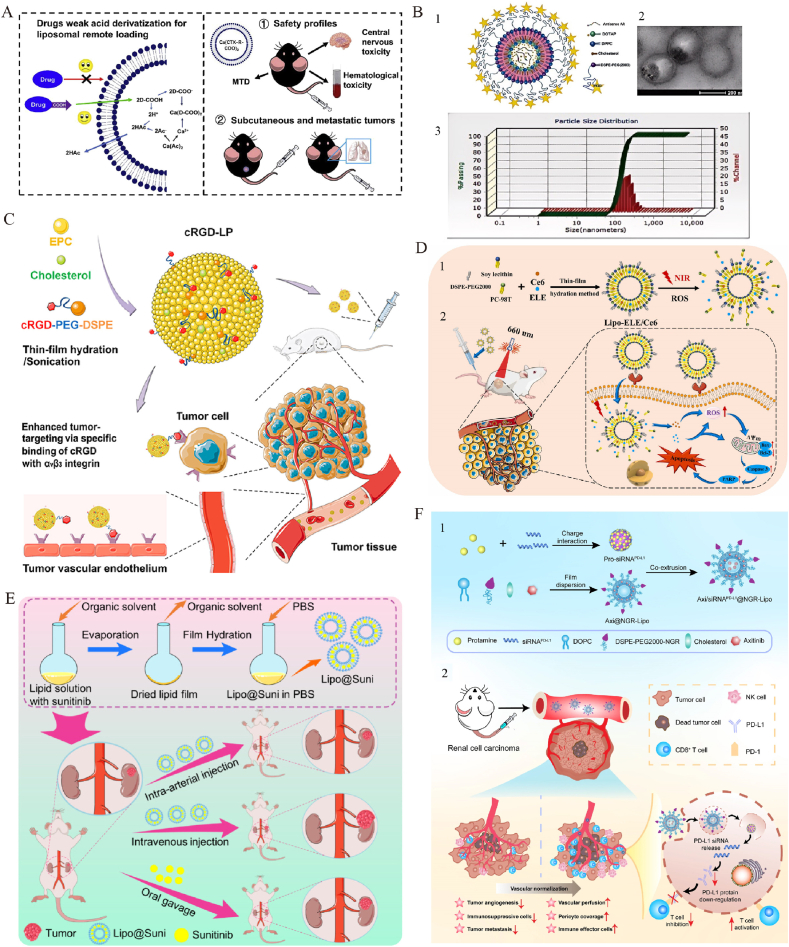
**Liposome-assisted therapy in urinary system tumors. A:** A facile and universal method was constructed to achieve liposomal remote loading of non-ionizable drugs. The developed cabazitaxel weak acid derivatives liposomes were superior to the marketed formulations, Jevtana®, concerning the safety profiles and antitumor efficiency. Reproduced with permission from Ref. [203]. **B:** iRGD-liposome-ASO synthesis, validation, and pharmacokinetics. (1) Schematic diagram of the iRGD-liposome-ASO system showing iRGD, antisense AR, and liposomes with lipid materials (DOTAP, DPPC, Cholesterol, and DSPE-PEG2000). (2) TEM image of final constructs of iRGD-liposome-ASO showing cloudy liposomal coatings around dark cores. Scale bar: 200 nm. 3) Hydrodynamic diameter and zeta potential of iRGD-liposome-ASO. Reproduced with permission from Ref. [204].**C:** The design and preparation of cRGD-decorated liposome (cRGD-LP) with enhanced tumor-targeting for drug delivery. Reproduced with permission from Ref. [205]. **D:** Schematic illustration of Lipo-ELE/Ce6 for combined photo-chemotherapy. (1) Construction of Lipo-ELE/Ce6; (2) Effect of Lipo-ELE/Ce6 + Laser on enhancing apoptosis of NMIBC. Reproduced with permission from Ref. [214]. **E** Schematic Illustration of Lipo@Suni Preparation and Its Different Routes of Administration (Intra-Arterial, Intravenous, and Oral Gavage) for RCC Treatment. Reproduced with permission from Ref. [224]. **F:** Construction of NGR peptide-modified actively targeted liposomes Axi/siRNAPD−L1@NGR-Lipo co-encapsulating Axi and siRNAPD−L1 and mechanism of combining anti-angiogenic and ICI therapies to enhance anti-tumor immunotherapy for the treatment of renal cell carcinoma. Reproduced with permission from Ref. [226].

Various modifications significantly enhance liposome targeting and therapeutic outcomes. Tian et al. [202] developed PSMA-antibody-conjugated liposomes co-delivering antisense oligonucleotides and oxaliplatin, effectively downregulating PI3K/AKT3 signaling and GLUT-1 expression, inhibiting tumor cell proliferation, migration, and inducing apoptosis. Addressing cabazitaxel's poor solubility, Zhou et al. [203] created weakly acidic derivatives loaded via remote pH-gradient methods, demonstrating superior antitumor effects compared to commercial Jevtana® in PCa and lung metastasis mouse models (Fig. 7A). Guan et al. [204] employed iRGD-modified liposomes to enhance AR-ASO accumulation in tumors, showing notable suppression of AR-V7 variants (Fig. 7B). Cyclic RGD peptide-modified liposomes delivering AT-101 overcame its poor oral bioavailability and toxicity issues, enhancing stability, controlled release, and targeted delivery (Fig. 7C) [205]. Cu(TPZ)_2-loaded liposomes targeting hypoxia via remote loading significantly increased solubility and cytotoxicity in 3D PCa spheroid models [206]. EGFR-targeted immunoliposomes carrying cabazitaxel displayed reduced particle size, high encapsulation efficiency, and superior cytotoxicity and antitumor effects, significantly extending survival with reduced toxicity in EGFR-overexpressing PCa models [207]. Mourtas et al. [208] synthesized novel ether-linked arsenolipid-containing liposomes exhibiting stable encapsulation and dose- and time-dependent anticancer effects, suggesting a promising approach for stable arsenic-based therapeutics. Another strategy delivered a perforin-expressing plasmid driven by PSA promoters using liposomes, significantly enhancing perforin expression and inhibiting tumor growth [209]. Lastly, RGD-modified nanoliposomes co-loaded with arsenic trioxide (ATO) and curcumin showed remarkable apoptosis induction (up to 98 %) and suppressed EGFR expression in PC3 cells, highlighting their synergistic antiproliferative efficacy [210].

#### 5.1.2 Innovations of liposomes in bladder cancer

Beyond generic nanocarrier attributes, liposomes offer a membrane-mimetic bilayer that accommodates mixed-polarity payloads and exhibits superior performance under intravesical dosing despite urine dilution and micturition [211]. Stimuli-responsive formulations—thermosensitive, pH/enzyme/ROS-triggered, and fusogenic or deformable (transfersomes)—permit on-site activation within the bladder wall and broaden the therapeutic window [212]. In combined chemo-immunotherapy, Pal et al. [213] developed reduction-sensitive oxaliplatin prodrug liposomes (LRO), further encapsulated with low-dose BCG and chitosan (LRO-BCG/CS), which induced immunogenic cell death (ICD) and immune activation, achieving strong tumor suppression with favorable safety in murine models. Zhang et al. [214] constructed a photoresponsive liposome (Lipo-ELE/Ce6) co-loaded with Ce6 and β-elemene, which synergistically triggered ROS generation and tumor apoptosis under laser irradiation, showing promising outcomes in NMIBC treatment (Fig. 7D).

For targeted therapy, ATF24-modified liposomes co-delivering cisplatin effectively targeted uPAR-positive bladder cancer cells, promoting apoptosis and cell cycle arrest [215]. Another study reported PEGylated β-elemene liposomes with improved pharmacokinetics, prolonged half-life, and enhanced antitumor efficacy [216]. EphA2 antibody-modified liposomes carrying paclitaxel prodrug (DTXp) precisely targeted EphA2-overexpressing tumors and outperformed free drugs in PDX models, underscoring their translational potential [217]. PEGylated cisplatin liposomes (PLCispt) developed by Ghaferi et al. [218] showed potent antitumor effects with lower systemic toxicity and improved controlled release.

Liposomes co-encapsulating ciprofloxacin and epirubicin significantly reduced cardiotoxicity without compromising efficacy, proposing a novel cardioprotective strategy for anthracycline-based therapies [219]. In immunotherapy, Lip-TDM liposomes formulated with BCG-derived trehalose dimycolate activated the Mincle–CD8^+^ T cell axis, achieving broad-spectrum anticancer effects with good biocompatibility [220]. Whang et al. [221] developed BCG-CWS liposomes that induced apoptosis via AMPK/JNK signaling, providing a safer alternative to live BCG. Additionally, hybrid membrane liposomes (3025@ML) co-delivered with oxaliplatin disrupted tumor metabolism by targeting PKM2 and FASN pathways, significantly enhancing apoptosis and expanding metabolic-targeted strategies in bladder cancer therapy [222].

#### 5.1.3 Liposomal formulations for synergistic RCC treatment

In renal cell carcinoma (RCC) treatment, numerous studies have explored liposome-based multifunctional nanocarrier systems by integrating anticancer small molecules, siRNA, and targeting peptides. These systems aim to overcome therapeutic challenges such as drug resistance, insufficient targeting, and systemic toxicity. For localized delivery of tyrosine kinase inhibitors like sunitinib, PEGylated liposomes (lipo-sunitinib) have been developed. This system not only induced tumor cell autophagy but also enhanced T cell and cDC1 infiltration, thereby activating systemic antitumor immunity and providing dual therapeutic benefits for RCC [223]. Another study employed intra-renal arterial injection of Lipo@Suni to elevate local drug concentration and retention time, enabling more precise delivery and improved efficacy (Fig. 7E) [224].

Resveratrol liposomes (RES-lips) combined with sorafenib inhibited the PI3K-AKT-mTOR and VHL-HIF pathways, effectively overcoming drug resistance and stimulating immune responses, showing potent antitumor effects in sorafenib-resistant models [225]. In anti-angiogenesis and immunotherapy, NGR-modified liposomes co-delivering axitinib and PD-L1 siRNA (Axi/siRNAPD−L1@NGR-Lipo) mitigated immune escape induced by targeted therapy, promoted immune cell infiltration, and reduced immunosuppressive cell populations, demonstrating strong synergistic antitumor potential (Fig. 7F) [226]. For radiosensitization, a dual-loaded liposome (EY-L) carrying everolimus and YM155 was developed, which enhanced radiotherapy by inhibiting DNA repair, downregulating cell cycle checkpoints, and inducing mitotic catastrophe, while reducing systemic toxicity [227].

#### 5.1.4 Applications of liposomes in other types of urinary system tumors

In testicular cancer, Lo et al. designed a PEGylated liposome co-loaded with hepcidin TH1-5 and epirubicin. This formulation significantly enhanced drug uptake and cytotoxicity in SCC15 squamous carcinoma and NT2D1 embryonal carcinoma cells. By suppressing P-gp and MRP expression, increasing ROS generation, and inducing mitochondrial apoptosis, the system effectively reversed multidrug resistance (MDR), marking the first demonstration of hepcidin-assisted chemosensitization via liposomal delivery [228].

### 5.2 Dendrimers

Dendrimers are a class of highly branched, three-dimensional, monodisperse synthetic macromolecules characterized by a tree-like architecture with numerous terminal functional groups on their surfaces. The internal core structure of dendrimers contains tunable porous cavities that create a unique microenvironment suitable for drug encapsulation and transport. By attaching appropriate targeting ligands to their surfaces, dendrimers can act as highly efficient and selective drug delivery vehicles capable of targeting specific cells while sparing normal tissues [229] (Table 10).

**Table 10:** Therapeutic applications of dendrimers in urologic tumors.

	Nanoparticle	Nanoparticle Size(nm)	Outcome	Cell Lines	Stage	Ref.
PC	PD-CTT1298-Cabo	4.2 ± 0.2	PSMA-targeted neutral PAMAM dendrimer delivers cabozantinib with enhanced efficacy and reduced toxicity.	PC3-PIP cells	in vitro	233
	PSMA-2DG-D-Cabo	4.5 ± 0.19	PSMA-2DG-D delivers cabozantinib for enhanced prostate cancer targeting and efficacy.	PC3-PIP cells	in vivo in vitro	234
	PAMAM-His-PEG-Trp	–	LHRH-targeted PAMAM nanoconstructs efficiently silence MCL-1 and inhibit tumor growth.	LNCaP cells	in vivo in vitro	235
	DAB-PEG-SS-ODT	260.8 ± 36.5	Self-assembling redox-sensitive PEG-DAB vesicles enable prostate cancer gene delivery.	PC-3,DU145 cells	in vitro	236
	scFv(AM1)-P-BAP-polyplexes	135.5	PSCA-targeted mPPI-siRNA polyplexes suppress Survivin and enhance prostate cancer therapy.	PC3,PC3PSCA cells	in vivo in vitro	237
	G2Ru	–	G2Ru dendrimer disrupts HIF-1α/VEGF signaling and blocks prostate cancer cell cycle.	LNFLU, LNCaP cells	in vitro	238
	2[G4]-N3 Dendrimer	3.1	Aptadendrimer efficiently delivers C8 and induces apoptosis in prostate cancer cells.	PNT1A, DU-145,PC-3 cell	in vitro	239
	Jeff-[G10a]_2_-SHa	174.1	Jeff-[G10a]_2_-SHa dendrimer shows dual anticancer-antibacterial activity with low hemolytic toxicity.	PC-3 cells	in vitro	240
BC	siNrf2-GCD	130	siNrf2-GCD delivery system overcomes CDDP resistance in bladder cancer with good safety and therapeutic efficacy.	T24,253J B-V, 253J B-V C-r cells	in vivo in vitro	243
	PEG-PAMAM-DOX	13	PEG-PAMAM-DOX enhanced intravesical DOX penetration and tumor-targeted release.	MB-49 cells,sv-huc-1 cells	in vivo in vitro	244
	SiPcPGal4	–	SiPcPGal4 improves PDT efficacy via galactose dendrons, enhancing uptake and targeting cancer cells effectively.	UM-UC-3 cells	in vitro	245

Dendrimers have been shown to significantly improve the pharmacokinetics, safety, and therapeutic efficacy of bioactive ligands and therapeutic agents [230,231]. Among them, poly(amidoamine) (PAMAM) dendrimers have been extensively studied for applications in cancer imaging, diagnostics, and therapy (Table 11). Their well-defined structure makes them especially suitable for multifunctional purposes, such as the simultaneous delivery of drugs and genes, or the co-delivery of multiple therapeutic agents for synergistic treatment [232].

**Table 11:** Diagnostic and theranostic applications of denderimers in urologic tumors.

	Nanoparticle	Nanoparticle Size(nm)	Outcome	Cell Lines	Stage	Ref.
PC	PT-DDC	5.3 ± 1.8	Multimodal PSMA-targeted dendrimer enhances prostate cancer therapy and imaging precision.	PC3 pip,PC3 flu cells	in vivo in vitro	241
	conjugateIV	5	PSMA-targeted PAMAM dendrimers enable high-resolution dual-mode FL/PA imaging of prostate cancer.	PC3 pip,PC3 flu cells	in vivo in vitro	242
RCC	Dendrimer nanoparticles	–	Multivalent nanosurface enables sensitive clinical capture of RCC-CTCs for liquid biopsy.	ACHN, 786-O cells	in vitro	247

#### 5.2.1 Applications of dendrimers in PCa

In recent years, extensive research has focused on dendrimer-based nanomedicines for prostate cancer, particularly in enhancing targeting specificity, delivery efficiency, and multifunctional regulation. To improve tumor targeting, PD-CTT1298, a prostate-specific membrane antigen (PSMA)-targeted platform, was developed by conjugating an irreversible PSMA ligand to a generation 4 hydroxyl-terminated PAMAM (PAMAM-G4-OH), enabling precise recognition and drug delivery to prostate cancer cells [233]. A dual-functional PSMA and 2-deoxy-D-glucose (2DG) dendrimer system (PSMA-2DG-D) further enhanced the tumor accumulation and efficacy of cabozantinib while reducing systemic toxicity [234].

Luteinizing hormone-releasing hormone (LHRH) peptide-modified PAMAM dendrimers effectively delivered anti-MCL-1 siRNA, inducing apoptosis and tumor suppression in LNCaP cells, thus offering a targeted strategy for LHRH-overexpressing tumors [235]. Another study constructed PEGylated G3-DAB-based redox-sensitive cationic nanovesicles for DNA delivery, which achieved controlled release under high glutathione conditions, providing an ideal vehicle for tumor gene therapy [236]. Maltose-modified poly(propylene imine) (mPPI) dendrimers targeting PSCA delivered BIRC5/Survivin siRNA, showing significant tumor inhibition in a mouse model [237].

**Fig. 8:**
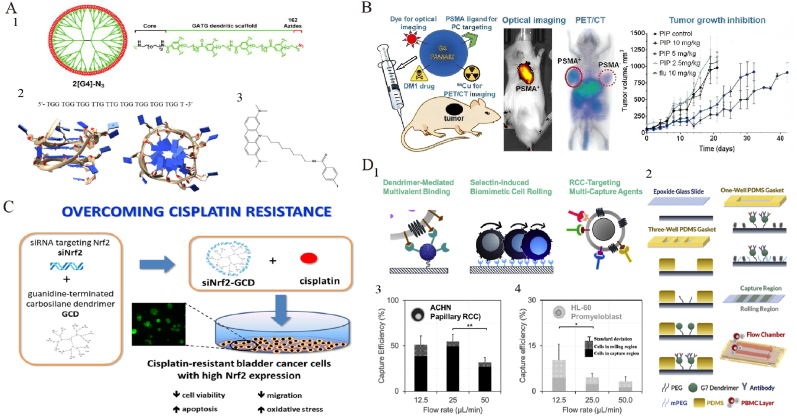
**Dendrimer-based nanoplatforms in urinary system tumors. A:** (1) Chemical structure of the GATG repeating unit. (2) 2[G4]-N3 dendrimer with 162 terminal azides. (3) G-rich nucleotide sequence and tridimensional structure of AT11 aptamer (PDB entry 2N3M) and (4) chemical structure of 10-(8-(4-iodobenzamide)octyl))-3,6-bis(dimethylamine) acridinium iodide, also known as C8. Reproduced with permission from Ref. [239].**B:** Multifunctional PAMAM-based theranostic system (PT-DDC) co-loaded with DM1, Cy5 dye, and ˆ64Cu-NOTA for prostate cancer targeting. The nanoprobe enables PSMA-specific drug delivery, optical and PET/CT imaging, and demonstrates significant tumor growth inhibition. Reproduced with permission from Ref. [242]. **C:** siNrf2-GCD system using guanidine-terminated carbosilane dendrimer (GCD) for siRNA delivery to overcome cisplatin resistance in bladder cancer by silencing Nrf2, enhancing cytotoxicity, and reducing viability, migration, and oxidative stress. Reproduced with permission from Ref. [243]. **D:** (1) Schematic of a microfluidic capture system for RCC-CTCs utilizing dendrimer-mediated multivalent binding, biomimetic rolling, and RCC-specific antibodies; (2) Fabrication steps of the capture and rolling domains in PDMS flow chambers; (3–4) Capture efficiency of RCC (ACHN) and control (HL-60) cells at different flow rates, with highest specificity observed at 25 μL/min. Reproduced with permission from Ref. [247].

A carboxyl-terminated silane dendrimer modified with a ruthenium-NHC complex (G2Ru) demonstrated inhibition of the HIF-1α/VEGF pathway, pro-apoptotic effects, and antioxidant activity, effectively suppressing castration-resistant prostate cancer [238]. An aptadendrimer combining the G-quadruplex aptamer AT11 and the anticancer ligand C8 facilitated cellular uptake and specific intracellular release for targeted prostate cancer therapy (Fig. 8A) [239]. Additionally, a multifunctional [G10a]-Temporin SHa dendritic peptide, especially its Jeffamine-modified version, exhibited enhanced in vivo stability and therapeutic efficacy, showing promise in both anticancer and antimicrobial applications [240].

In prostate cancer theranostics, Lesniak et al. developed a PSMA-targeted dual-modal imaging platform using fourth-generation PAMAM dendrimers, capable of fluorescence and photoacoustic imaging. By tuning the number of conjugated dyes, they achieved improved imaging contrast and specificity with minimal off-target signals in PSMA-positive tumor models, offering potential for intraoperative guidance [241]. Building on this, a multifunctional PAMAM-drug conjugate system (PT-DDC) was developed, co-delivering the microtubule inhibitor DM1, fluorescent dye Cy5, and radiolabeling chelator NOTA. This system demonstrated excellent stability, controlled drug release, and high PSMA specificity, integrating therapy, fluorescence imaging, and nuclear tracking into a single platform for precise and visible prostate cancer treatment (Fig. 8B) [242].

#### 5.2.2 Dendrimer-based drug platforms in bladder cancer and RCC therapy

Recent studies have developed various dendrimer-based drug delivery systems to enhance the efficacy of conventional chemotherapy and photodynamic therapy (PDT) for bladder cancer. To address cisplatin resistance, a system based on amine-modified carbosilane dendrimers (GCD) delivering Nrf2 siRNA (siNrf2-GCD) was designed. This platform effectively downregulated the expression of the resistance-related gene Nrf2, thereby restoring cisplatin sensitivity while demonstrating excellent cellular uptake and biosafety profiles (Fig. 8C) [243].Another study introduced a PEG-PAMAM-modified doxorubicin delivery system (PEG-PAMAM-DOX) capable of penetrating the bladder barrier and releasing the drug under acidic tumor microenvironments. In an orthotopic bladder cancer mouse model, it exhibited potent localized antitumor activity, highlighting its potential as an intravesical chemotherapy vehicle for postoperative treatment [244]. In the field of PDT, a novel benzophenazine-core silicon phthalocyanine derivative (SiPcPGal_4_) bearing four galactose units was synthesized. This photosensitizer demonstrated high water solubility, efficient cellular uptake, and potent phototoxicity in bladder cancer cells, showing promise as a next-generation targeted PDT agent [245].

In renal cancer, dendrimer platforms enable high-valency presentation of RCC-relevant ligands and synergize with biomimetic cell rolling to sensitively capture heterogeneous CTCs, conferring unique advantages for liquid-biopsy diagnostics and theranostics [246]. A resulting multifunctional dendrimer system achieved >5-fold higher CTC detection sensitivity in clinical samples, offering a practical tool for early RCC diagnosis and disease monitoring (Fig. 8D) [247].

### 5.3 Poly (lactic-co-glycolic acid)

Poly(lactic-co-glycolic acid) (PLGA), composed of polylactic acid (PLA) and polyglycolic acid (PGA), is a biodegradable and biocompatible polymer whose properties can be finely tuned by altering the PLA-to-PGA ratio [248]. This adjustability affects degradation rate, hydrophobicity, and drug release kinetics, making PLGA a customizable platform for therapeutic applications. Its key advantages in nanomedicine include excellent mechanical strength, programmable biodegradability, and the capacity to encapsulate a broad range of therapeutic agents, including chemotherapeutics, RNA, and proteins [249].PLGA's ability to provide controlled and sustained drug release enhances drug solubility, stability, and bioavailability, thereby overcoming several limitations associated with conventional chemotherapy. In the treatment of genitourinary cancers, PLGA-based nanoparticles have been employed to encapsulate drugs such as bicalutamide, improving their stability and promoting targeted delivery to cancer cells. This not only reduces systemic side effects but also increases drug accumulation at tumor sites, improving therapeutic outcomes (Table 12). Additionally, PLGA nanoparticles can be functionalized with targeting ligands or imaging agents, enabling theranostic applications that integrate diagnosis and therapy [250].

**Table 12:** Applications of PLGA in urinary system tumors.

	Nanoparticle	Nanoparticle Size(nm)	Outcome	Cell Lines	Stage	Ref.
PC	DNA-scaffolded PLGA NPs	209	DNA-scaffolded ligand tuning enhanced PSMA-targeted nanoparticle uptake by ∼3-fold with superior specificity.	PC3 pip,PC3 flu cells	in vivo in vitro	251
	PLGA-myricetin	–	Myricetin inhibits KDM4, enhances cytotoxicity, and synergizes with enzalutamide for better tumor suppression.	C4-2B cells	in vitro	252
	PLGA-PEG copolymer	209.4 ± 2.7	Engineered PSMA-targeted Brusatol/docetaxel nanoparticles synergistically enhanced prostate cancer therapy.	LNCaP,PC-3	in vitro	253
	Di-PP/AR-siRNA/DTX	101.4	PSMA-targeted nanosystem co-delivers DTX and AR siRNA, significantly enhancing CRPC therapeutic efficacy.	LNCaP cells	in vivo in vitro	254
	PLGA-ICG-R848 NPs	157.7	Developed ICG-R848 dual-functional PLGA nanoparticles achieving photothermal-immunotherapy synergy.	PC-3,LNCaP,DU-145 cells	in vivo in vitro	255
	DOX@H-PEG-CSPNs	137.8	Developed acidity-triggered dePEGylated micelles enhancing tumor uptake and doxorubicin efficacy.	TRAMP-C1 cells	in vivo in vitro	256
	T CBZ NPs	191.8 ± 0.473	ALN-modified PLGA NPs significantly inhibit bone metastatic prostate cancer progression and reduce side effects.	PC3,C4-2B cells	in vitro	257
	ZA/PEI@PBCTPNs	132.9 ± 7.4	PLGA stably encapsulates ZA and enables pH-triggered release for enhanced prostate cancer therapy.	TRAMP-C1 cells	in vivo in vitro	258
	PSMA-PLGA	475.9	Developed PSMA-targeted PLGA nanocarriers enabling high-efficiency nucleic acid encapsulation and gene therapy.	22Rv1,LNCaP cells	in vitro	259
	GEF@PLGA NPs	61.10 ± 1.97	PLGA as core material stabilizes drug loading and controls ZA release, enhancing tumor delivery and antitumor efficacy.	22RV1 cells	in vivo in vitro	260
	ICG nanobubbles	353.8 ± 153.1	Enhancing photoacoustic signal and GRPR targeting, offering potential for clinical prostate cancer imaging.	PC3-GFP, DU145-GFP cells	in vivo in vitro	261
	PLGA-b-PEG-NH2	160	Engineered PSMA-targeted PLGA NPs for boron delivery and imaging, yet rapid boron release limits BNCT efficacy.	PC3-flu, PC3-pip cells	in vivo in vitro	262
BC	WGA PLGA 503H NPs	326.7 ± 2.9	PLGA nanoparticles with WGA surface modification enhance bladder drug delivery and cell adhesion.	SV-HUC cells	in vitro	263
	CS-CBD/PLGA NPs	287.20 ± 0.90	CBD induces bladder cancer cell apoptosis via PI3K/Akt pathway.	T24,5637,UM-UC-3 cells	in vivo in vitro	264
	GemC14-PLGA-NP	215.5 ± 15.2	N-acylation of gemcitabine improves lipophilicity; PLGA nanoparticles with WGA enhance bladder cancer therapy.	5637 cells	in vitro	265
	LBL PLGA NBs	298 ± 12	Enzyme-driven PLGA nanobots enable urea-mediated swarming, enhancing pDNA delivery and transfection.	MB49 cells	in vitro	266
	si-m/hVDAC1-B	–	VDAC1 silencing reprograms metabolism and tumor microenvironment, suppressing bladder cancer progression.	UM-UC3,HTB-5 cells	in vivo in vitro	267
RCC	CZ-PLGA-NPs	210 ± 40	Inhibiting renal cancer metastasis through controlled release and immune modulation, showing adjuvant potential.	L929 cells	in vitro	268
	PEG-PLGA NPs	77.8 ± 0.5	Engineered LC-conjugated NPs selectively target kidney and RCC via megalin, enabling safe and precise delivery.	HK-2 cells	in vivo in vitro	269
	ZnPP@G-PP NPs	286.9 ± 0.096	G250-modified PLGA nanoplatform enhances targeted SDT and real-time imaging for precise RCC theranostics.	786-O cells	in vivo in vitro	270

#### 5.3.1 PLGA nanoparticles for prostate cancer: sustained release and biocompatibility

**Fig. 9:**
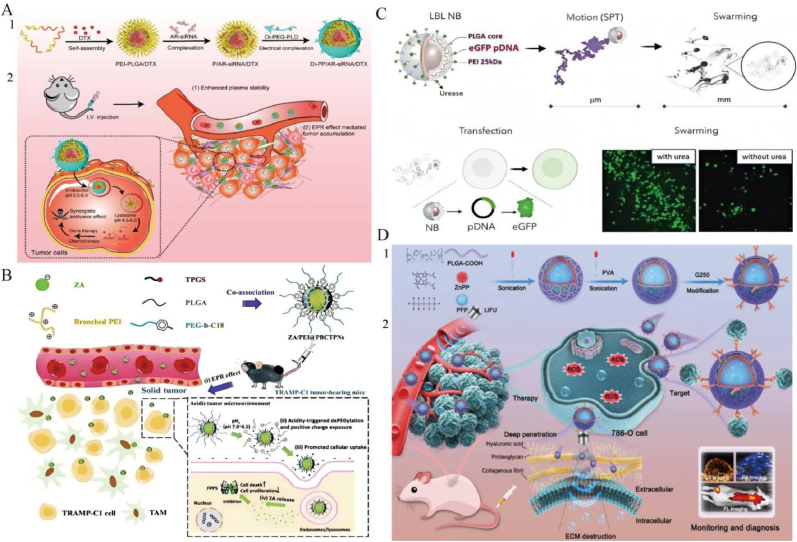
**PLGA-mediated drug delivery in urinary system tumors. A:** Illustration of the preparation and the mechanism of Di-PP/AR-siRNA/DTX. (**1**) The formation of Di-PP/AR-siRNA/DTX. (**2**) The mechanism of anti-tumor activity of DTX and AR siRNA. Reproduced with permission from Ref. [254]. **B:** Illustration of promoted intratumoral accumulation and cellular uptake of tumor acidity-responsive ZA-carrying hybrid nanoparticles upon acid-triggered PEG detachment and positive charge exposure to enhance antitumor efficacy. Reproduced with permission from Ref. [258]. **C:** Construction of urease-powered, layer-by-layer nanorobots (NBs) for enhanced gene transfection. The nanorobots improve pDNA delivery efficiency, especially in 3D bladder cancer models, outperforming conventional transfection reagents. Fluorescence imaging confirms eGFP expression in MB49 cells 24 h post-transfection (scale bar: 300 μm). Reproduced with permission from Ref. [266]. **D:** Schematic illustration of ZnPP@G-PP NP synthesis (1) and the efficient accumulation of ZnPP@G-PP NPs in 786-O tumor cells, achieving targeted penetration and multimodal imaging-guided diagnosis and monitoring under sonodynamic action (2). Reproduced with permission from Ref. [270].

PLGA nanoparticles are increasingly recognized as critical carriers for integrated therapy and diagnosis of prostate cancer, owing to their versatile drug delivery modes, surface-targeting modifications, and imaging-enhancing capabilities. In therapeutic applications, PLGA-based platforms have been employed to deliver chemotherapeutics, natural compounds, gene agents, and immunomodulators, enhancing antitumor efficacy while minimizing toxicity. By precisely tuning the density and spatial arrangement of PSMA ligands, researchers have developed highly efficient PSMA-targeted PLGA nanoparticles that significantly improve cellular uptake in prostate cancer cells [251]. To overcome drug resistance, natural compound myricetin was loaded into PLGA nanoparticles (PLGA-MYR) and co-administered with enzalutamide, resulting in enhanced antitumor effects [252]. PSMA-targeted PLGA-PEG nanoparticles have also enabled the co-delivery of brusatol and docetaxel, promoting ROS generation and tumor suppression [253]. The PEI-PLGA system facilitates the co-delivery of docetaxel and AR siRNA, achieving synergistic chemogene therapy, particularly suitable for castration-resistant prostate cancer (CRPC) (Fig. 9A) [254]. For combined photothermal and immunotherapy, PLGA-ICG-R848 nanoparticles induce potent thermal ablation under near-infrared light and activate dendritic and NK cells, achieving dual-modality treatment [255]. Acid-responsive onion-like PLGA micelles (DOX@H-PEG-CSPNs) expose a cationic layer upon PEG detachment in acidic environments, enhancing cellular uptake and cytotoxicity [256]. In addressing bone metastatic prostate cancer, ALN-modified PLGA nanoparticles have been used to deliver cabazitaxel to bone sites, effectively inhibiting tumor growth and metastasis [257]. Similarly, zoledronic acid-loaded acid-sensitive PLGA nanoparticles demonstrated potent local antitumor effects, particularly suited for bone-associated lesions (Fig. 9B) [258]. In gene therapy, PLGA systems encapsulating siRNA combined with PSMA-617 targeting showed markedly improved gene silencing efficiency [259]. PLGA also improved the solubility and tumor accumulation of poorly bioavailable drugs such as gefitinib (GEF) [260].

In diagnostic and theranostic applications, PLGA demonstrated promise through the development of ICG-loaded PLGA nanobubbles conjugated with GRPR-targeting ligands, enabling enhanced photoacoustic/ultrasound dual-modal imaging in mouse models of prostate cancer [261]. Moreover, Meher et al. synthesized PSMA-targeted PLGA-b-PEG nanoparticles for boron neutron capture therapy (BNCT) and PET imaging. Although rapid boron release remains a challenge, the study provides a foundation for novel radiotherapeutic strategies [262].

#### 5.3.2 Innovations of PLGA in bladder cancer and RCC

In the treatment of bladder cancer, PLGA nanoparticles have been employed to enhance the efficacy and targeting of conventional therapeutics. Brauner et al. developed PLGA nanoparticles encapsulating wheat germ agglutinin (WGA) and trifluoromethylphenothiazine (TMP), which significantly improved mucoadhesion to the bladder urothelium and prolonged drug retention, offering a promising strategy for intravesical drug delivery [263]. Additionally, a chitosan-coated CBD-PLGA nanoparticle system was designed to deliver cannabidiol (CBD), a photosensitizer with potent pro-apoptotic effects on bladder cancer cells, enhancing therapeutic outcomes via improved bladder adhesion [264]. A modified N-acylated gemcitabine-PLGA nanoparticle system was shown to improve drug lipophilicity and cellular uptake, markedly enhancing antitumor efficacy in bladder cancer models [265]. PLGA also demonstrates distinct advantages in gene therapy. A self-propelling, layer-by-layer assembled nanobot (NB) system significantly improved plasmid DNA transfection efficiency, particularly in 3D bladder cancer cell models, outperforming conventional transfection agents (Fig. 9C) [266]. Similarly, PLGA-PEI nanoparticles delivering Nrf2-siRNA successfully overcame cisplatin resistance in bladder cancer, enhancing the drug's cytotoxicity [267].

In renal cancer therapy, Lee et al. [268]. formulated PLGA nanoparticles encapsulating cabozantinib (CZ), a multi-target tyrosine kinase inhibitor, to address its poor oral bioavailability and low water solubility, thereby improving its therapeutic efficacy and reducing renal cancer cell proliferation and migration Moreover, PLGA nanoparticles conjugated with WGA improved targeting to renal tubular epithelial cells, enabling precise drug delivery to renal cancer cells [269]. For theranostic applications, PLGA-based platforms also show great promise. A ZnPP@G-PP PLGA nanoparticle system was designed to combine ultrasound-targeted drug delivery (UTMD) with sonodynamic therapy (SDT) for clear cell renal cell carcinoma (ccRCC). Upon activation by low-intensity focused ultrasound, the system generated reactive oxygen species (ROS) and enabled trimodal imaging (photoacoustic, fluorescence, and ultrasound) for real-time localization and therapeutic monitoring, significantly enhancing drug delivery efficiency and treatment efficacy (Fig. 9D) [270].

### 5.4 Others

In addition, various emerging nanodelivery platforms — such as exosomes, extracellular vesicles (EVs) and nanogels— have demonstrated considerable potential in drug delivery, image-guided therapy, and radio-/immunosensitization for urological malignancies.

Exosomes and EVs are endogenous lipid vesicles that preserve donor-cell membrane proteins and glycosylation patterns, featuring low immunogenicity, natural homing ability, and efficient transmembrane transport. They can encapsulate nucleic acids, small molecules, and proteins, and their membranes can be engineered via ligand modification or membrane hybridization to enhance targeting specificity while maintaining stability in complex biofluids [271]. In the urinary system, urinary exosomes serve as non-invasive liquid biopsy carriers for detecting and stratifying biomarkers of prostate, bladder, and renal cancers [272]. Therapeutically, immune- or mesenchymal stem cell-derived EVs can deliver siRNA/miRNA or immune-regulatory molecules to modulate the tumor microenvironment and improve chemo-/radiosensitivity. Moreover, EVs can be combined with adhesive biomaterials to prolong intravesical retention and enhance tissue uptake [273].

Nanogels are hydrophilic polymeric three-dimensional networks that exhibit injectability, in situ gelation, and programmable release properties. They can be triggered by temperature, pH, enzymatic activity, or reactive oxygen species (ROS), while offering strong mucoadhesiveness, tunable mechanics, and biodegradability [274]. In urological applications, thermosensitive or adhesive nanogels can markedly extend intravesical drug retention, resist urinary dilution, and achieve sustained local delivery at high concentrations with minimal systemic exposure [275]. “Hydrogel–nanoparticle hybrid depots” combining chemotherapeutics, immunoadjuvants, or nanocarriers have been applied for postoperative consolidation therapy and recurrence prevention in NMIBC [276]. Additionally, nanogels spacers in prostate radiotherapy have clinically proven to reduce rectal radiation dose, and similar strategies are being extended to renal and bladder postoperative cavities for localized drug delivery and tissue repair [277].

## 6 Hybrid nanoparticles

Hybrid nanoparticles (HNPs) are nanoscale structures composed of at least two distinct types of nanomaterials, designed to overcome the limitations of single-component nanoparticles, enhance performance, or achieve multifunctionality beyond the capability of individual nanostructures [278]. Featuring diverse architectures such as core–shell, yolk–shell, and Janus structures, HNPs hold tremendous potential for applications in drug delivery and cancer therapy [279]. In the context of urologic oncology, practically relevant HNP classes include lipid–polymer hybrids (e.g., PLGA core with lipid shell), membrane-coated/biohybrid systems (e.g., cancer- or immune-cell membranes cloaking inorganic or polymeric cores), and inorganic–organic composites such as MSN–lipid or MOF–polymer assemblies (Table 13 . Compared to traditional monocomponent nanoparticles, HNPs offer several advantages, including multifunctional integration, improved biocompatibility, and enhanced targeting efficiency. In oncology, HNPs have garnered increasing attention for their ability to simultaneously enable drug loading, targeted delivery, image guidance, and combination therapy through the rational integration of distinct functional nanomaterials. By co-locating high loading (MSN), prolonged circulation/immune evasion (lipid or membrane cloak), and ligand/antibody targeting, HNPs integrate drug loading, targeting, image guidance and combination therapy, with thermo/pH/enzyme/ROS-triggered release to widen the therapeutic window [280,281]. Another key advantage of HNPs is their capacity for multimodal imaging—incorporating MRI, optical imaging, and fluorescence imaging—which facilitates accurate tumor diagnosis and real-time therapeutic monitoring [282] (Table 14). In hybrid nanoparticle platforms (HNPs), structural complementarity enables parallel enhancement of microenvironment modulation, immune activation, and external physical triggering [283,284]. For example, membrane-coated ferrite–drug hybrids activate the cGAS–STING pathway and remodel the immune microenvironment to strengthen antitumor efficacy [285]

**Table 13:** Therapeutic applications of HNPs in urologic tumors.

	Nanoparticle	Nanoparticle Size(nm)	Outcome	Cell Lines	Stage	Ref.
PC	AuNCs-DAB-Lf	92.65 ± 0.57	Developed AuNCs-DAB-Lf-DNA for efficient TNFα delivery and PC-3 inhibition without external stimuli	PC-3 cells	in vitro	286
	AuNP-DOX/Fab	30	Dendronized AuNPs enable acid-triggered DOX release and EphA2-targeted prostate cancer therapy.	PC3 cells	in vitro	287
BC	FA-TMLs@MNPs-GNRs-DOX	230	Achieving 95 % drug release and 93 % cytotoxicity in bladder cancer cells upon laser activation.	5637,A549 cells	in vitro	297
	Dox@MSNPs-G2	124.5 ± 3.7	EnhancING mucoadhesiveness and achieve pH-responsive Dox release.	UMUC3 cells	in vivo in vitro	298
RCC	SLB-MPNPs	285.9	PLGA-based magnetic nanocarriers enable sustained release and targeted anticancer effects of Silibinin.	A-498 cells	in vivo in vitro	303

**Table 14:** Diagnostic and theranostic applications of HNPs in urologic tumors.

	Nanoparticle	Nanoparticle Size(nm)	Outcome	Cell Lines	Stage	Ref.
PC	Lipo-gold	96 ± 20	Engineered platform enables synergistic radiosensitization and CT imaging for image-guided cancer therapy	PC-3 cells	in vitro	288
	APT-PEG-Au-MMNPs@ELC	81.13 ± 7.41	Developed an EpCAM-targeted pH-responsive platform for efficient cancer targeting and reduced systemic toxicity.	PC-3,CHO cells	in vivo in vitro	289
	Gd2O3-MS NSs	95 ± 1.5	Gd2O3-MS NSs enable dual T1/T2 MRI imaging and photothermal therapy for “see and treat” applications.	–	in vitro	290
	LF-bis-MPA-MNPs	66.5 ± 17	Enabling rapid, high-yield EV isolation and sensitive urinary miRNA detection.	–	in vitro	291
	AuMNPs	400	A dual-enhanced SERS immuno-nanocomplex enables multiplex PSA detection and PHI assessment	–	in vitro	292
	Ag/IO-GRP	100	Dual-function magnetoplasmonic detection and purification-free exosome detection.	–	in vitro	293
	Fe3O4@ TMU-10	–	The detection limit of 0.45 pg/mL shows its potential for clinical applications.	–	in vitro	294
	MMIP,PSA@DTNB@Au	18–22	The sensor shows excellent selectivity and sensitivity with a detection limit of 0.9 pg/mL.	–	in vitro	295
	M-SiO2@NAC-CuNCs	250.0	Developed a highly sensitive “turn off-on” detection platform for ACP activity.	–	in vitro	296
BC	MNs@12-peptide,AgNPs@Ab	50	Developed a dual-enhancement SERS platform for UBC detection with a limit of 6.25 ng/mL,	A375 cells	in vitro	299
	MAGIC	63.3 ± 7.1	MAGIC nanoparticles show significantly reduced serum protein adsorption, optimizing sensitivity.	–	in vitro	300
	Janus Magnetic Microspheres	–	A multiplex detection platform utilizing structural color encoding and magnetic responsiveness enrichment.	T24 cells	in vitro	301
	Au@4-MPBA@HA/Fe3O4@DTNB@Au@MEA	400	A self-calibration SERS platform based on “core-satellite” structure achieves highly sensitive and accurate detection of HAase.	–	in vitro	302
RCC	Mn-MoS2 QDs	6.07	AS1411-Mn-MoS2 QDs enable efficient dual-modal imaging and fluorescence labeling of renal cell carcinoma.	786-O rena cells	in vivo in vitro	304
Others	Fe3O4NPs@COF/AuNPs	600–700	Dual nanostructures enable ultrasensitive electrochemical AFP sensing	–	in vitro	305
	Ag-DTNB-DOTA-MIBG	1081	Dual-targeted SERS platform enables sensitive recognition and magnetic isolation of PCC-CTCs.	PC12 cells	in vitro	306
	AgNPs/PAMAM/GCE	–	Constructed sensor enables dynamic O2•^−^ detection in living cells with low LOD and high reproducibility.	PC12 cells	in vitro	307

### 6.1 Multifunctional hybrid platforms in prostate cancer theranostics

In recent years, hybrid nanoparticle platforms (HNPs) have shown significant promise in the diagnosis and treatment of prostate cancer. The AuNCs-DAB-Lf system, which co-modifies gold nanocages (AuNCs) with lactoferrin and dendritic polymers, greatly improves DNA loading and transfection efficiency, enabling efficient gene delivery without external stimulation and enhancing TNF-α–mediated antiproliferative activity, highlighting its potential in gene therapy [286]. Another study developed a polypropylenimine (PPI)-modified gold nanoparticle platform with pH-sensitive doxorubicin release and conjugation to an EphA2 antibody fragment. This system achieved tumor microenvironment-responsive delivery and potent cytotoxicity in PC-3 cells (IC_50_ as low as 0.9 nM) [287].

**Fig. 10:**
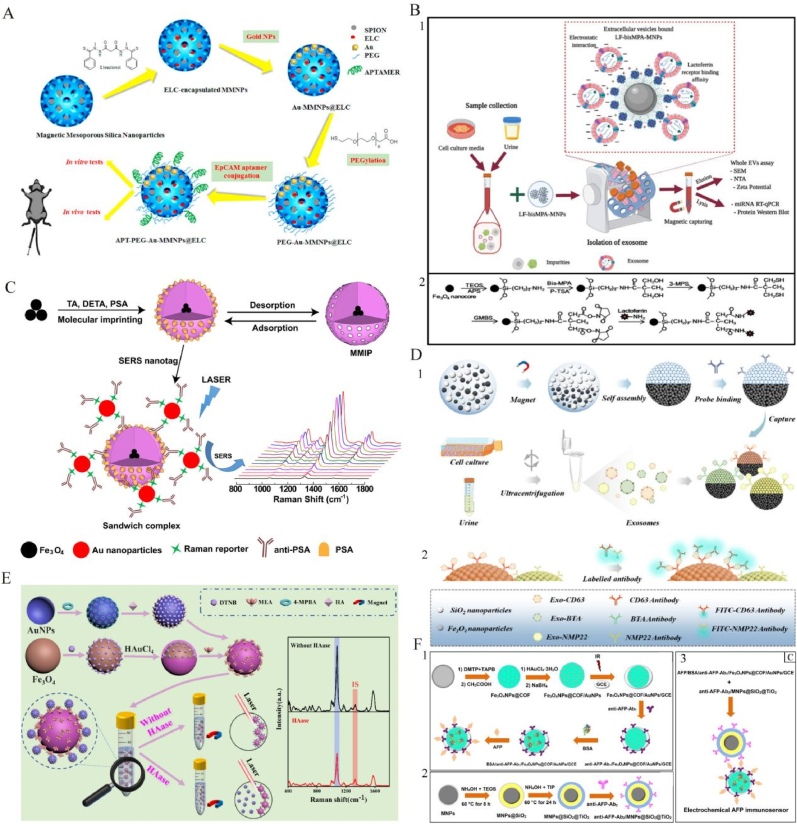
**Multifunctional HNP strategies in urinary system tumors. A:** Schematic representation of the ELC-loaded gold-silica nanoparticles. Reproduced with permission from Ref. [289].**B:** A schematic illustration and the mechanism of EV isolation by LF-*bis*-MPA-MNPs. (a) Schematic diagram of the LF-*bis*-MPA-MNPs for EV isolation and detection in CCM and human urine (created with BioRender.com). (b) Schematic diagram of synthesis and modification of the LF-*bis*-MPA-MNPs. Reproduced with permission from Ref. [291].**C:** Schematic presentation for fabrication of plasmonic sensor. Reproduced with permission from Ref. [295].**D:** Schematic diagram of the Janus magnetic microspheres for noninvasive analysis of bladder cancer-derived urinary exosomes. (a) Preparation of the Janus magnetic microspheres and the immobilization of antibody probes for exosome identification and capture and (b) multiplex exosome analysis platform based on the Janus magnetic microsphere barcodes. Reproduced with permission from Ref. [301].**E:** Schematic diagram of HAase detection using the “core-satellite” model SERS sensing platform. Reproduced with permission from Ref. [302].**F:** Schematic illustration of the fabrication procedure of electrochemical AFP immunosensor. Reproduced with permission from Ref. [305]. (For interpretation of the references to color in this figure legend, the reader is referred to the Web version of this article.)

The combination of multiple functional materials has accelerated the development of integrated theranostic platforms. For example, Lipogold—gold nanoparticles coated onto liposomes—offers drug loading, controlled degradation, photothermal responsiveness, and radiosensitization, enhancing radiotherapy sensitivity and providing strong CT imaging contrast for image-guided radiotherapy (IGRT) [288]. The APT-PEG-Au-MMNPs@ELC platform, incorporating gold nanoparticles, mesoporous silica, and EpCAM aptamers, enables pH-responsive drug release and tumor-specific targeting, effectively suppressing tumor growth in vitro and in vivo while reducing systemic toxicity (Fig. 10A) [289]. Moreover, the Gd_2_O_3_-MS NSs platform, composed of gadolinium oxide and gold-coated mesoporous silica, supports T1/T2 dual-mode MRI and near-infrared photothermal therapy (PTT), offering real-time temperature feedback and multimodal imaging for precision theranostics [290].

The development of multifunctional HNPs has also advanced non-invasive and highly sensitive prostate cancer diagnostics. Magnetic nanomaterials, due to their enrichment ability and imaging capabilities, are widely used in biomarker detection. A lactoferrin-functionalized dendritic magnetic nanoparticle system (LF-bis-MPA-MNPs) enables rapid exosome isolation from urine, significantly improving the sensitivity and specificity of miRNA biomarker detection (e.g., miR-21, miR-346), aiding urine-based screening for prostate and bladder cancers (Fig. 10B) [291]. In serum biomarker detection, noble metal–magnetic hybrid nanostructures such as gold-silver nanoflowers combined with magnetic gold nanoparticles have enabled multiplex quantification of fPSA, cPSA, and p2PSA, enhancing diagnostic accuracy in patients within the PSA “gray zone” via the Prostate Health Index (PHI) [292]. A graphene platform modified with silver–iron oxide nanoparticles and fluorescent antibody labeling achieved high-sensitivity exosome detection without purification, simplifying traditional workflows [293].

Beyond conventional immunoassays, MOF–quantum dot composites have been introduced for enzyme-free biosensing. For example, a ferromagnetic MOF electrode combined with nickel-doped CdTe quantum dots enabled ultrasensitive and stable PSA detection (LOD = 0.45 pg/mL) [294]. Additionally, magnetic molecularly imprinted polymers (MMIPs) built with tannic acid allowed antibody-free PSA recognition, achieving a SERS-based detection limit of 0.9 pg/mL and offering a cost-effective diagnostic strategy (Fig. 10C) [295]. Finally, a Cu nanocluster-loaded M − SiO_2_ platform, coupled with MnO_2_ in an on–off fluorescence system, enabled sensitive quantification of ACP, a potential early marker of prostate cancer [296].

### 6.2 Hybrid nanoparticles for drug delivery in bladder cancer and RCC

For bladder cancer therapy, Songwei et al. developed a folate receptor-targeted, magneto-thermo-photoresponsive drug delivery system (FA-TMLs@MNPs-GNRs-DOX). Using a microfluidic chip, Fe_3_O_4_ magnetic nanoparticles, photothermal gold nanorods (GNRs), and doxorubicin (DOX) were co-encapsulated into folate-modified thermosensitive liposomes. Drug release was triggered by near-infrared (NIR) irradiation, significantly enhancing tumor targeting and cytotoxicity [297]. Another study employed PAMAM-modified mesoporous silica nanoparticles (MSNPs) with pH-responsive properties, improving local drug retention and mucosal adhesion in the bladder, offering an effective intravesical therapy vehicle [298].

Multifunctional hybrid platforms combining magnetic, metallic, and quantum components have notably improved diagnostic sensitivity and specificity. Hou et al. developed a sandwich SERS biosensor for urinary bladder cancer antigen (UBC) detection using a peptide-antibody pair on magnetic and silver nanoparticles. Magnetic enrichment and SERS amplification achieved a detection limit of 6.25 ng/mL, enabling early, noninvasive screening [299]. The MAGIC nanoplatform, with a poly-CBMA polymer coating, allowed pg/mL-level biomarker detection while showing superior biocompatibility over traditional PEG and PEI materials [300]. Wei et al. designed Janus magnetic microspheres with structural color encoding and magnetic enrichment for high-throughput urinary exosome analysis, facilitating efficient and multiplexed liquid biopsy (Fig. 10D) [301]. Chen et al. introduced a core–satellite Fe_3_O_4_–Au nanostructure for ratiometric SERS detection of hyaluronidase (HAase) with a limit of detection of 0.32 mU/mL, validated in urine samples for early bladder cancer diagnosis (Fig. 10E) [302].

For renal cancer, Takke et al. used magnetic core-shell SLB-MPNPs to encapsulate silibinin in PLGA, enabling sustained release over 15 days and potent inhibition of A-498 cells with good biocompatibility [303]. Additionally, AS1411 aptamer-modified Mn-MoS_2_ quantum dots offered enhanced MRI/fluorescence dual-modality imaging, with high specificity toward renal cancer cells, representing a promising platform for multimodal diagnosis [304].

### 6.3 Applications of HNPs in other types of urinary system tumors

Hybrid nanoparticles (HNPs) have also been extensively applied in other urologic tumors. For testicular cancer diagnosis, Bölükbaşi et al. developed an electrochemical AFP immunosensor combining Fe_3_O_4_ NPs@COF/AuNPs electrodes with a dual-shell magnetic nanoparticle signal amplifier (MNPs@SiO_2_@TiO_2_), achieving ultra-sensitive detection of AFP with a detection limit of 3.30 fg/mL (Fig. 10F) [305]. In adrenal cancer, particularly pheochromocytoma (PCC), HNPs have demonstrated high sensitivity and multifunctionality. Meng et al. [306] constructed a SERS dual-targeting platform using Ag nanocubes and Fe_3_O_4_ nanoparticles to detect PCC circulating tumor cells (CTCs) in peripheral blood. Ag@DTNB enhanced Raman signals, while Fe_3_O_4_ conjugated with MIBG and DOTA enabled efficient magnetic enrichment, reaching a detection limit of 1 cell/mL, highlighting its promise for early liquid biopsy. Another study developed a non-enzymatic electrochemical sensor using PAMAM dendrimers loaded with Ag nanoparticles to monitor superoxide anion (O_2_•^-^) release from live cells. This platform offered high sensitivity, broad linearity, and stability, overcoming limitations of enzyme-based ROS detection and providing a novel tool to assess oxidative stress and tumor microenvironment regulation in PCC [307].

## 7 Discussion

### 7.1 Application of nanodelivery systems

In recent years, nanoparticle-based delivery systems have demonstrated remarkable advantages in the diagnosis and treatment of urologic malignancies, emerging as a research hotspot in cancer therapy. Rapid advances in nanotechnology have enabled precise drug delivery, with breakthroughs in enhancing drug targeting, improving solubility, prolonging circulation time, and reducing toxicity [308,309]. By surface modification and ligand conjugation, nanoparticles can efficiently target tumor cells, minimizing damage to normal tissues [310].

**Fig. 11:**
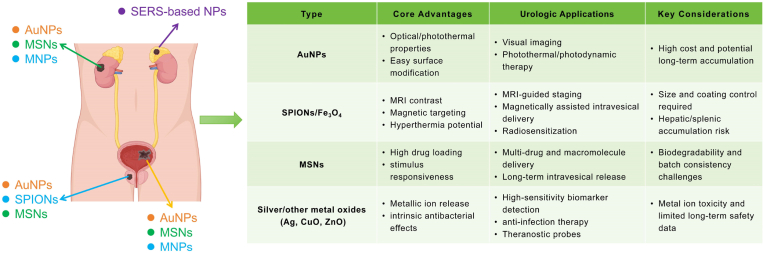
Comparative overview of inorganic nanoparticles and their site-specific applications in urologic tumors.

In inorganic nanocarrier systems, different materials exhibit distinct functional divisions in terms of physicochemical properties and drug delivery pathways. Gold nanoparticles (AuNPs), leveraging their excellent plasmonic resonance and tunable surface chemistry, are well suited for integrated imaging–guided photothermal and photodynamic platforms [58]. Magnetic nanoparticles (SPIONs/MNPs) primarily serve as MRI contrast agents and enable magnetic targeting and hyperthermia, making them ideal for urologic applications that require preoperative or intraoperative localization and externally guided intravesical delivery [118]. Mesoporous silica nanoparticles (MSNs), characterized by high surface area and customizable pore structures, demonstrate superior loading capacity and stimulus responsiveness, making them suitable for macromolecule co-delivery, multidrug systems, or sustained local release [163]. In contrast, silver and other metallic or metal oxide nanoparticles are more frequently applied in high-sensitivity detection, antibacterial and antitumor synergy, and integrated theranostic sensing platforms [101] (Fig. 11).

**Fig. 12:**
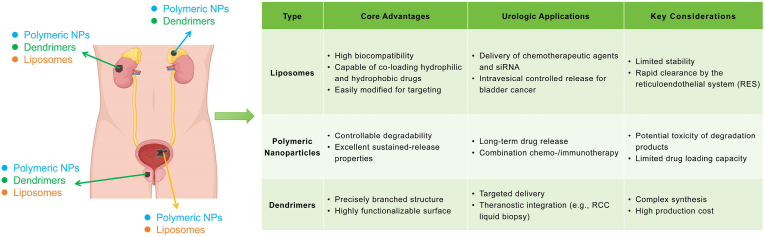
Comparative overview of organic nanoparticles and their site-specific applications in urologic tumors.

In organic nanocarrier systems, liposomes utilize a cell membrane–like bilayer structure to enable co-loading and controlled release of both hydrophilic and hydrophobic drugs. They represent the most clinically mature platform, widely applied in the delivery of chemotherapeutic agents, nucleic acids, and immunoadjuvants [188]. Dendrimers, characterized by their precisely branched architecture and multifunctional terminal groups, enable multivalent targeting and efficient encapsulation of nucleic acids or macromolecular drugs, holding particular promise for theranostic (diagnostic–therapeutic) integration platforms [229]. Polymeric nanoparticles (e.g., PLGA or PCL systems) exhibit excellent stability and tunable degradability, making them suitable for sustained release and multidrug combination therapies [248] (Fig. 12).

### 7.2 Biodegradability and long-term toxicity of nanomaterials

At present, nanodelivery systems face biocompatibility and long-term toxicity issues across multiple biological aspects. Immuno-safety is a primary concern, the in vivo protein corona reshapes nanoparticle “biological identity,” heightening innate recognition, triggers complement activation–related pseudoallergy (CARPA) and cytokine release, and—via anti-PEG antibodies—causing accelerated clearance and efficacy variability [311,312]. Second, organ accumulation and chronic injury: prolonged RES uptake (liver, spleen, marrow) drives organelle damage, oxidative stress, and fibrosis; some inorganic/metallic systems release ions that induce hepatorenal dysfunction and geno-/reprotoxicity [313]. Third, hemocompatibility and vascular safety: inappropriate size/charge/roughness can provoke hemolysis, platelet activation, and coagulation perturbation [314]. Additionally, degradation byproducts—especially from metallic carriers—may elicit hypersensitivity and organ toxicities, including hepatic–splenic deposition, thrombosis, immune activation, and local inflammation [315]. Further attention is warranted for dose- and population-level heterogeneity,immune memory with repeat dosing, drug interactions, impurities/endotoxin and residual solvents, and batch variability—can amplify adverse events during long-term follow-up [316].

In recent years, many studies have advocated an integrated design philosophy of “biodegradability–excretion–low residual burden” [[317], [318], [319]]. Accordingly, introducing controllably degradable linkages or soluble/inorganic backbones and finely tuning particle size/morphology/stiffness to promote renal or hepatobiliary clearance, together with surface engineering (zwitterionic/neutral stealth layers, switchable de-PEG strategies, and protein-corona modulation) and preferential local or intraluminal administration with thermo/enzymatic/ROS triggers to reduce systemic exposure, can effectively mitigate the long-term toxicity of nanodelivery systems.

### 7.3 Pathways to clinical translation

Clinical translation of nanodelivery systems remains constrained by biocompatibility, long-term toxicity, and reproducibility of efficacy. Most programs are preclinical or early-phase, with few reaching phase III or approval. In genitourinary cancers, clinically promising arenas include imaging-based stratification, locally heat-triggered release, and radiosensitization—for example, Ferumoxtran-10 for presurgical nodal staging in prostate cancer (phase III) [320], intraprostatic HfO_2_ nanoparticles (NBTXR3) as a radiosensitizer [321], LTLD plus local hyperthermia in bladder cancer [322], and CRLX101 combined with anti-angiogenic therapy in metastatic RCC [323]. PEGylated liposomal doxorubicin (Doxil/Caelyx, approved in 1995) [191] and the LNP–siRNA patisiran (approved in 2018) [324] have demonstrated established efficacy and safety in clinical practice, underscoring the translational potential of nanodelivery.

Key barriers to clinical translation include [325,326]: (1) the complexity of animal-to-human dose extrapolation and exposure assessment—variability in the EPR effect, population heterogeneity, and differences in release kinetics complicate starting-dose selection and dosing intervals; (2) immunogenicity and interindividual variability, which drive exposure fluctuations and uncertain tolerability; and (3) scale-up and CMC consistency challenges, whereby CQAs may drift with process changes, alongside stringent requirements for stability, endotoxin/residual-solvent control, and method harmonization.

### 7.4 Future directions and outlook

**Fig. 13:**
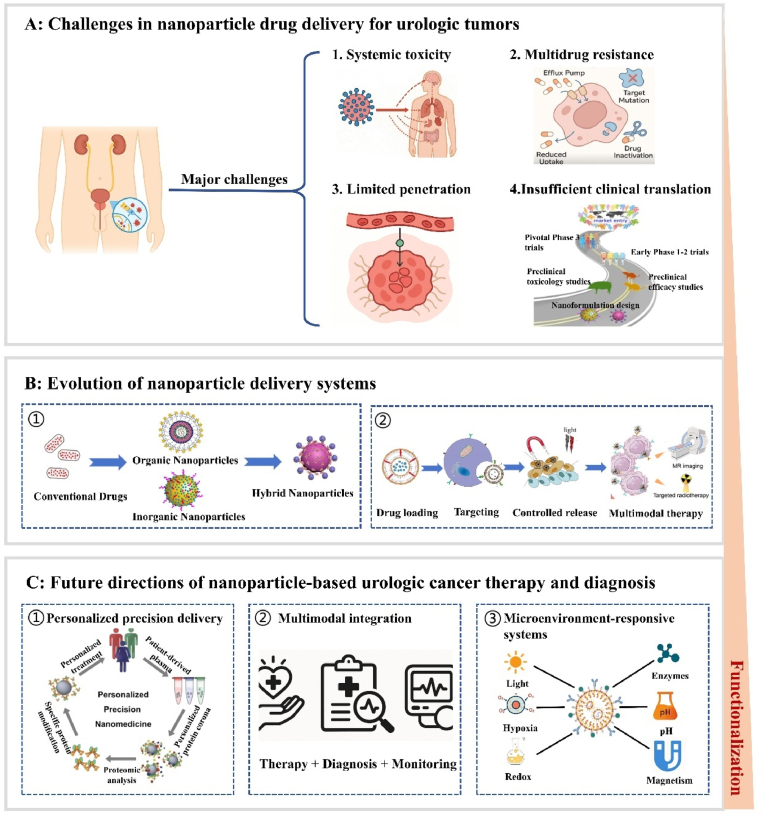
**Challenges, evolution, and future directions of nanoparticle-based drug delivery systems for urologic cancers. A:** Major challenges in current nanoparticle drug delivery include systemic toxicity, multidrug resistance, limited tumor penetration, and insufficient clinical translation. Reproduced with permission from FigDraw. **B:** Nanoparticle delivery systems have evolved from conventional drugs to organic, inorganic, and hybrid nanoparticles, along with advancements in drug loading, targeting, controlled release, and multimodal therapeutic strategies. Reproduced with permission from Ref**.** [335]**. C:** Future directions emphasize personalized precision delivery guided by omics data, integration of diagnosis, therapy and monitoring, and smart release systems responsive to tumor microenvironmental cues such as pH, enzymes, hypoxia, redox status, light, and magnetism. Reproduced with permission from ref**.** [336].

Looking ahead, the clinical potential of nanoparticle delivery systems remains promising but requires concerted effort on multiple fronts [327,328]. Research must continue to explore novel materials with higher drug-loading capacity, better stability, and optimized release kinetics. Integrated treatment strategies combining nanoparticles with chemotherapy, radiotherapy, and immunotherapy should be further investigated to maximize synergy. Future development of nanoparticle drug-delivery systems should place greater emphasis on personalized nanomedicine, structured around a “patient stratification–vector matching–dynamic evaluation” workflow: nanocarriers are selected according to tumor molecular markers and receptor expression profiles (e.g., PSMA, uPAR, EphA2) to achieve precise targeting and efficient delivery [329]. In parallel, advances in gene editing and nucleic-acid delivery make small-batch, patient-specific formulations increasingly feasible [330]. Emerging studies also highlight the use of nanomicellar systems for therapeutic cancer vaccine development and immunomodulatory applications, offering new insights into the integration of nanomedicine with immunotherapy and targeted therapy [331,332]. However, broad implementation remains constrained by cost, standardization and CMC consistency, and regulatory pathways [333]. Accordingly, future studies should further strengthen the clinical translation pathway by adopting a Quality by Design (QbD) paradigm, building an integrated nonclinical-to-clinical evidence chain anchored to critical quality attributes (CQAs), employing risk-tiered, phased trials with stratified enrollment, and incorporating real-world evidence (RWE) to continuously evaluate long-term safety and benefit—thereby advancing nanoparticle therapeutics toward individualized, manufacturable, and regulatory-compliant clinical implementation [334] (Fig. 13).

In conclusion, while nanoparticle delivery systems hold great promise in the diagnosis and treatment of urologic cancers, several key hurdles remain before widespread clinical adoption. With continued interdisciplinary collaboration and technological innovation, nanomedicine is poised to revolutionize precision oncology and offer patients safer, more effective, and personalized therapeutic options.

## CRediT authorship contribution statement

**Jiayi Ma:** Writing – review & editing, Writing – original draft. **Youlong Hai:** Writing – review & editing, Writing – original draft. **Kun Zheng:** Writing – review & editing, Writing – original draft. **Xiaoyong Hu:** Writing – review & editing, Supervision. **Kai Ni:** Writing – review & editing, Validation, Supervision, Investigation, Funding acquisition, Conceptualization.

## Ethics approval and consent to participate

Not applicable.

## Consent for publication

Not applicable.

## Availability of data

Not applicable.

## Fundings

This work was supported by the 10.13039/501100001809National Natural Science Foundation of China [grant number 82103260 to K.N.]; the Shanghai Rising-Star Program [grant number 22QA1407100 to K.N.]; the Excellent Youth Cultivation Program of Shanghai Sixth People's Hospital [grant number ynyq202204 to K.N.]; the Fundamental Research Funds of Shanghai Sixth People's Hospital (grant number: X-2490 to K.N.); the Fundamental Research Funds of the Shanghai Sixth People's Hospital (grant number: ynms202405 to Xiaoyong Hu).

## Declaration of competing interest

The authors declare that they have no known competing financial interests or personal relationships that could have appeared to influence the work reported in this paper.

## Data Availability

No data was used for the research described in the article.
